# Global Mapping of the Macrophage-HIV-1 Transcriptome Reveals that Productive Infection Induces Remodeling of Host Cell DNA and Chromatin

**DOI:** 10.1038/s41598-017-05566-9

**Published:** 2017-07-12

**Authors:** Alexandre Deshiere, Charles Joly-Beauparlant, Yann Breton, Michel Ouellet, Frédéric Raymond, Robert Lodge, Corinne Barat, Marc-André Roy, Jacques Corbeil, Michel J. Tremblay

**Affiliations:** 1 Axe des Maladies Infectieuses et Immunitaires, Centre de Recherche du Centre Hospitalier Universitaire de Québec-Université Laval, Québec, Canada; 2 Institut de Recherches Cliniques de Montréal, Montréal, Québec Canada; 3 Département de médecine moléculaire, Faculté de médecine, Université Laval, Québec, Canada; 4 Département de microbiologie-infectiologie et immunologie, Faculté de médecine, Université Laval, Québec, Canada

## Abstract

It has been proposed that macrophages could serve as long-lived compartments for HIV-1 infection under *in vivo* situations because these cells are resistant to the virus-mediated cytopathic effect, produce progeny virus over extended periods of time and are localized in tissues that are often less accessible by treatment. Comprehensive experimental studies are thus needed to characterize the HIV-1-induced modulation of host genes in these myeloid lineage cells. To shed light on this important issue, we performed comparative analyses of mRNA expression levels of host genes in uninfected bystander and HIV-1-infected human macrophages using an infectious reporter virus construct coupled with a large-scale RNA sequencing approach. We observed a rapid differential expression of several host factors in the productively infected macrophage population including genes regulating DNA replication factors and chromatin remodeling. A siRNA-mediated screening study to functionally identify host determinants involved in HIV-1 biology has provided new information on the virus molecular regulation in macrophages.

## Introduction

While the subject is still open for debate, some experimental and clinical studies have suggested that many key features of macrophages (MØ) could allow them to constitute a viral sanctuary in the context of HIV-1 infection^1–4^. For instance, MØ are more resistant to HIV-1-induced cell death^5^, display a longer lifespan than CD4^+^ T cells, and also possess self-renewal properties^6^. Indeed, HIV-1-infected MØ exhibit a greater telomerase activity^7^, an event associated frequently with chromosome maintenance and cellular longevity. Altogether, these findings suggest an inherent capability of MØ to produce HIV-1 over extended periods of time while showing minimal cell death associated with infection, all of which represent hallmarks of viral sanctuaries. A recent study performed in various humanized mouse models has revealed the ability of tissue-resident MØ to sustain and propagate HIV-1 infection under *in vivo* situations independently of CD4^+^ T cells^8^.

The evolution of HIV-1 has resulted in a multifaceted relationship with the intracellular environment where a plethora of host-encoded factors can regulate several steps of the virus life cycle. Expression of these cellular components is modulated by the activity of diverse soluble factors produced in response to viral infection. Reciprocally, different HIV-1 accessory proteins such as Nef, Vif, Vpu and Vpr (Vpx for HIV-2) control expression or activity of some specific host restriction and susceptibility factors but can also modify cellular behaviors in many ways^9^. In myeloid lineage cells, reverse transcription of HIV-1 genetic material is kinetically altered in comparison to activated CD4^+^ T cells. This phenomenon has been linked to lower cytoplasmic concentrations of deoxynucleotides associated with a high expression of the host restriction factor SAMHD1^10, 11^. More recently, numerous cellular factors closely linked to the type-I interferon (IFN) pathway have been shown to interfere with multiple steps of HIV-1 biology in myeloid cells. These include BST2/tetherin, IFIT, IFITM, TRIM and APOBEC families of proteins^12^. Nevertheless, there is paucity of data about the complexity of intracellular biochemical events leading to successful HIV-1 replication in MØ due, amongst other things, to the difficulty of discriminating between cells productively infected from those that remain uninfected (also called bystanders).

Therefore, we compared the HIV-1-induced transcriptional changes in productively infected and bystander human MØ. Our results reveal new information on the type-I IFN-related response in MØ by demonstrating that most factors associated with this pathway are expressed independently of virus infection. Our work also reveals the complexity of transcriptional changes at the level of cellular DNA and chromatin, which are occurring very early after productive HIV-1 infection. A selection of fifty genes, which expression levels were differentially modified in productively infected and bystander MØ when compared to uninfected cells, was evaluated in a siRNA screening assay and some host factors were found to regulate virus replication. Overall our observations indicate that HIV-1 infection affects host DNA and chromatin in human MØ.

## Results

### Efficient enrichment and sorting of productively infected and bystander MØ

**Figure 1:**
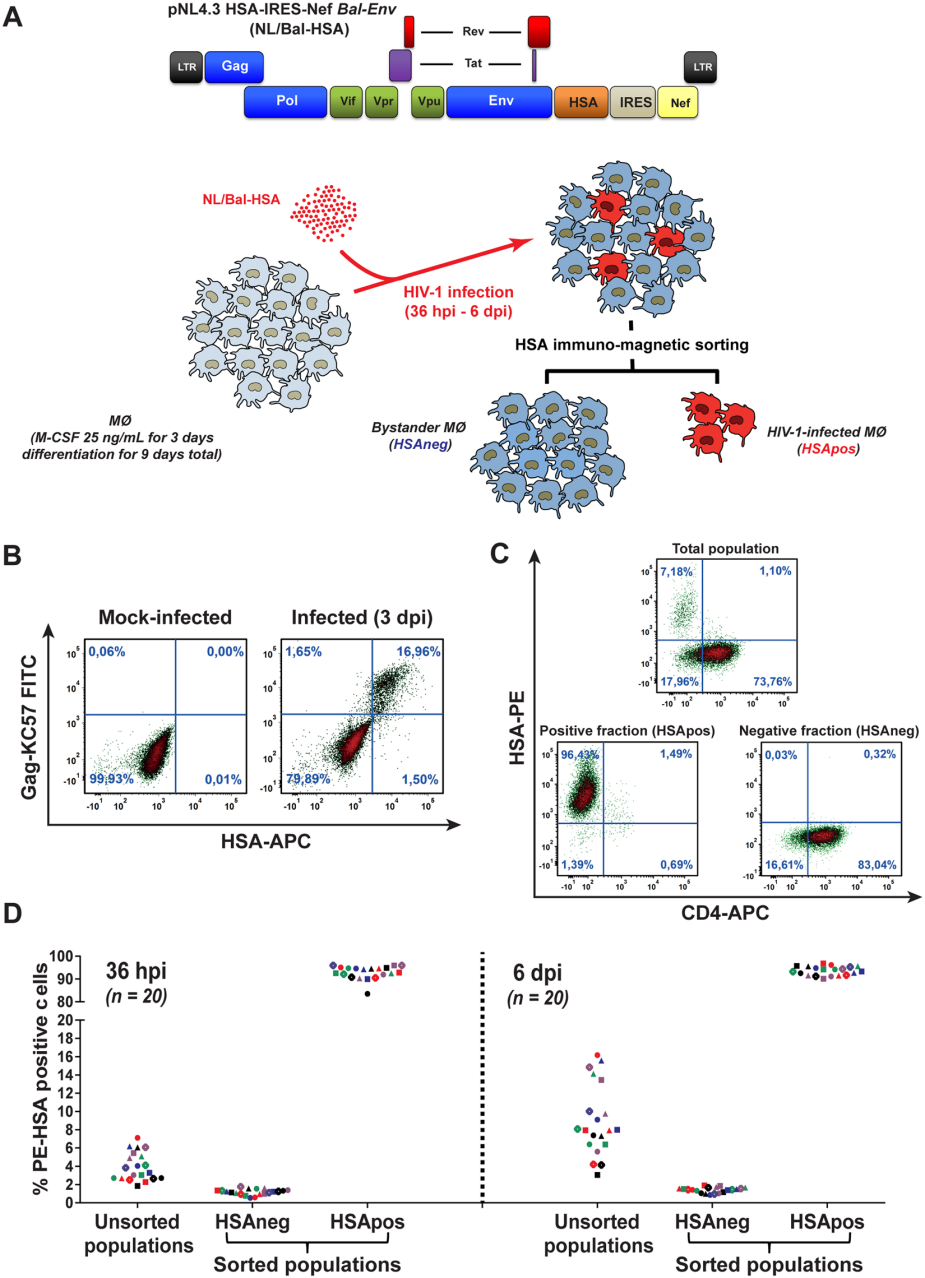
Efficient purification of HIV-1-infected and bystander MØ. (**A**) The murine *HSA* reporter gene was transcriptionally fused to the virus-encoded *Nef* gene to engineer the infectious molecular clone NL/Bal-HSA. MØ are first infected with NL/Bal-HSA reporter virus followed by an HSA-based immunomagnetic sorting to isolate and purify HSApos and HSAneg MØ. (**B**) Surface staining of the HSA epitope correlates with Gag expression (KC57 antibody) in flow cytometry analyses at 6 dpi. Data shown are representative of two replicate experiments. (**C**) Surface staining of the HSA epitope correlates with CD4 depletion at 6 dpi. Data are from a representative experiment of two replicate experiments. (**D**) HSApos MØ are efficiently enriched by the HSA-based immunomagnetic sorting process independently of the initial HIV-1 infection rate at 36 hpi or 6 dpi. MØ preparations from 20 distinct donors were used for these studies.

We developed a distinctive experimental system to isolate, enrich and discriminate between human MØ productively infected with HIV-1 and bystanders. This procedure relies on acute HIV-1 infection with a fully competent R5-tropic reporter virus coding for the murine surface molecule HSA (NL4.3 HSA-IRES-Nef *Bal-env*; called NL/Bal-HSA hereafter)^13, 14^ coupled with an immunomagnetic sorting method (Fig. 1A). The technical approach was validated by showing the presence of HIV-1 Gag in the purified HSApos cell population (Fig. 1B). Moreover our data demonstrate that HSApos MØ are CD4 negative and bystanders (HSAneg) expressed high levels of CD4, thus confirming that HSApos MØ are productively infected with HIV-1 (Fig. 1C). The purity of the HSApos MØ population was significantly increased by the enrichment step (ranging from 85 to 98%) and HSAneg MØ were efficiently depleted from HSApos cells (less than 2% of contamination) (Fig. 1D). The overall permissiveness of MØ preparations from different donors (a total of 20) to HIV-1 infection was highly variable between the different donors processed, ranging from 1 to 7% at 36 hours post-infection (hpi) and 3 to 17% at 6 days post-infection (dpi) (Fig. 1D). A dose-dependent increase in the number of HSApos MØ was observed with higher viral doses and peaks of virus infection were reached at around 6 dpi (see Supplementary Fig. S1A and B). Western blot analyses indicated that HIV-1 Gag-derived proteins were expressed and significantly enriched in HSApos MØ (see Supplementary Fig. S1C). Although a weak signal corresponding to the p24 capsid protein could be detected in the HSAneg population at 72 hpi (see Supplementary Fig. S1C), a quantitative PCR analysis of HIV-1 transcripts displayed negative levels of Tat mRNA (see Supplementary Fig. S1D).

### Large-scale remodeling of host gene expression in productively infected MØ

**Figure 2:**
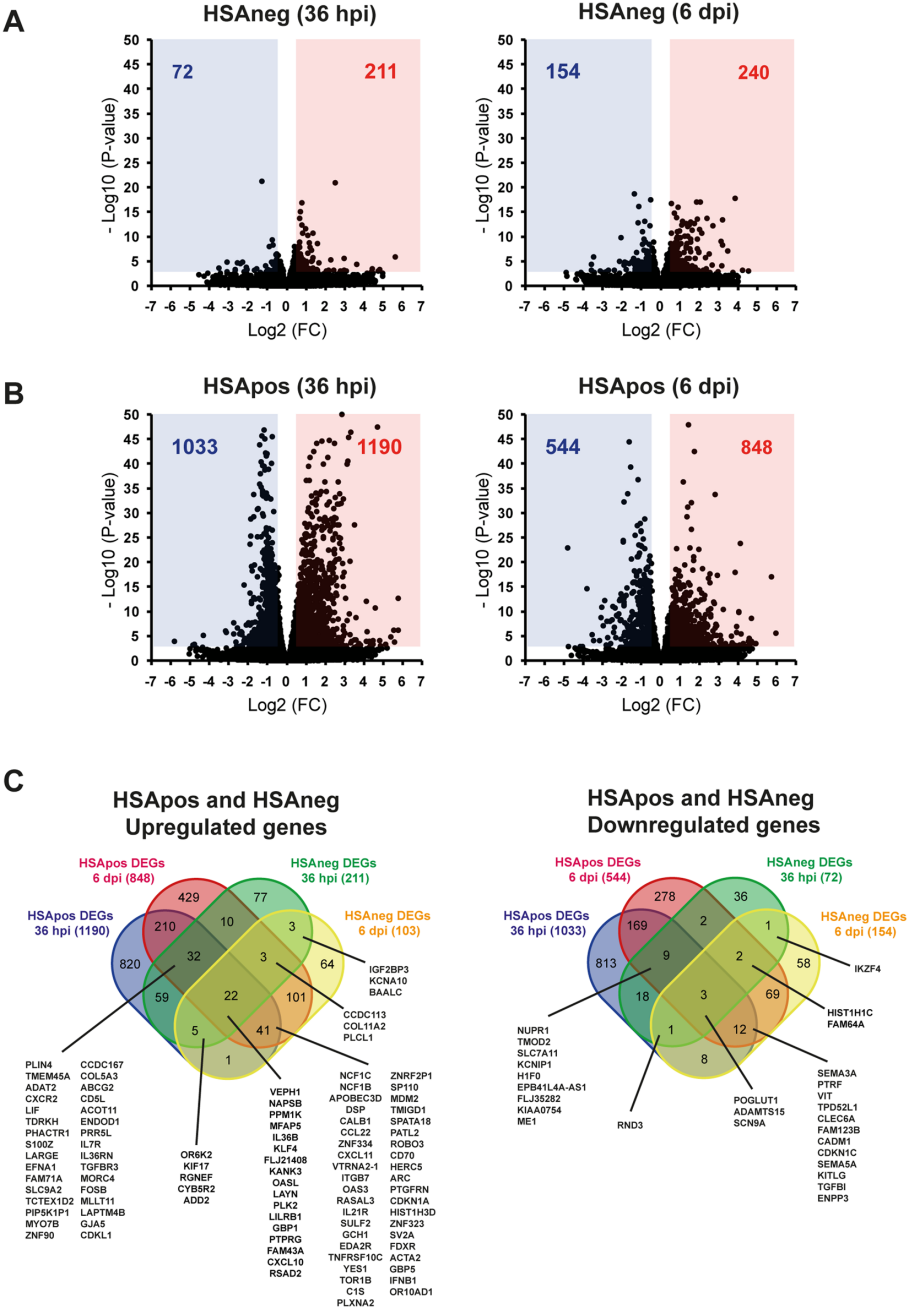
HIV-1 induces major changes in the MØ transcriptome very early after productive infection. Volcano plot analyses indicate that HSAneg MØ (**A**) are weakly affected by HIV-1 compared to the HSApos cell population (**B**) in RNAseq samples at 36 hpi and 6 dpi. Data are presented as Log2 Fold-Change (Log2 FC) of fragments per kilobase of transcript per million mapped reads (FPKM) with associated *p* values calculated from four independent experiments. (**C**) A comparison of Differentially Expressed Genes (DEGs) in HSApos and HSAneg MØ using a Venn diagram analysis shows that the regulation of the cellular environment is time-dependent in infected and bystander cells. The numbers indicate the total number of genes in each area. Diagrams were generated ﻿using﻿ the following tool: http://bioinformatics.psb.ugent.be/webtools/Venn/. The complete list of DEGs is supplied in the Supplementary Table S3.

To delineate the HIV-1-induced gene modulation in MØ, we performed RNA sequencing (RNAseq) analyses of the purified HSApos and HSAneg fractions. Figure 2 displays volcano plot representations of differentially expressed genes (DEGs) in both HSApos and HSAneg MØ for which we could detect significant fold changes in the mRNA expression levels when compared to mock-infected cells (log2 FC > 0.5; *p* value < 0.01). A much lower transcriptional modulation was detected in HSAneg (Fig. 2A) compared to HSApos MØ (Fig. 2B), especially at 36 hpi. For example, comparison between mock-infected and HIV-1-infected MØ revealed 2223 DEGs at 36 hpi and 1392 at 6 dpi, whereas only 283 DEGs were found in bystanders at 36 hpi, increasing to 394 at 6 dpi. A Venn diagram analysis for the different conditions is depicted in Fig. 2C and a complete list of DEGs can be found as Supplementary Table S1 (all DEGs) and S2 (DEGs after cutoffs values were applied). In the HSApos population, 210 host genes were up-regulated at both 36 hpi and 6 dpi and only 3 DEGs were increased at the two time points tested in the HSAneg MØ. Interestingly, several DEGs associated with type-I IFN, such as CXCL10, OASL, OAS3 and APOBEC3D, were enhanced in both HSApos and HSAneg populations.

**Figure 3:**
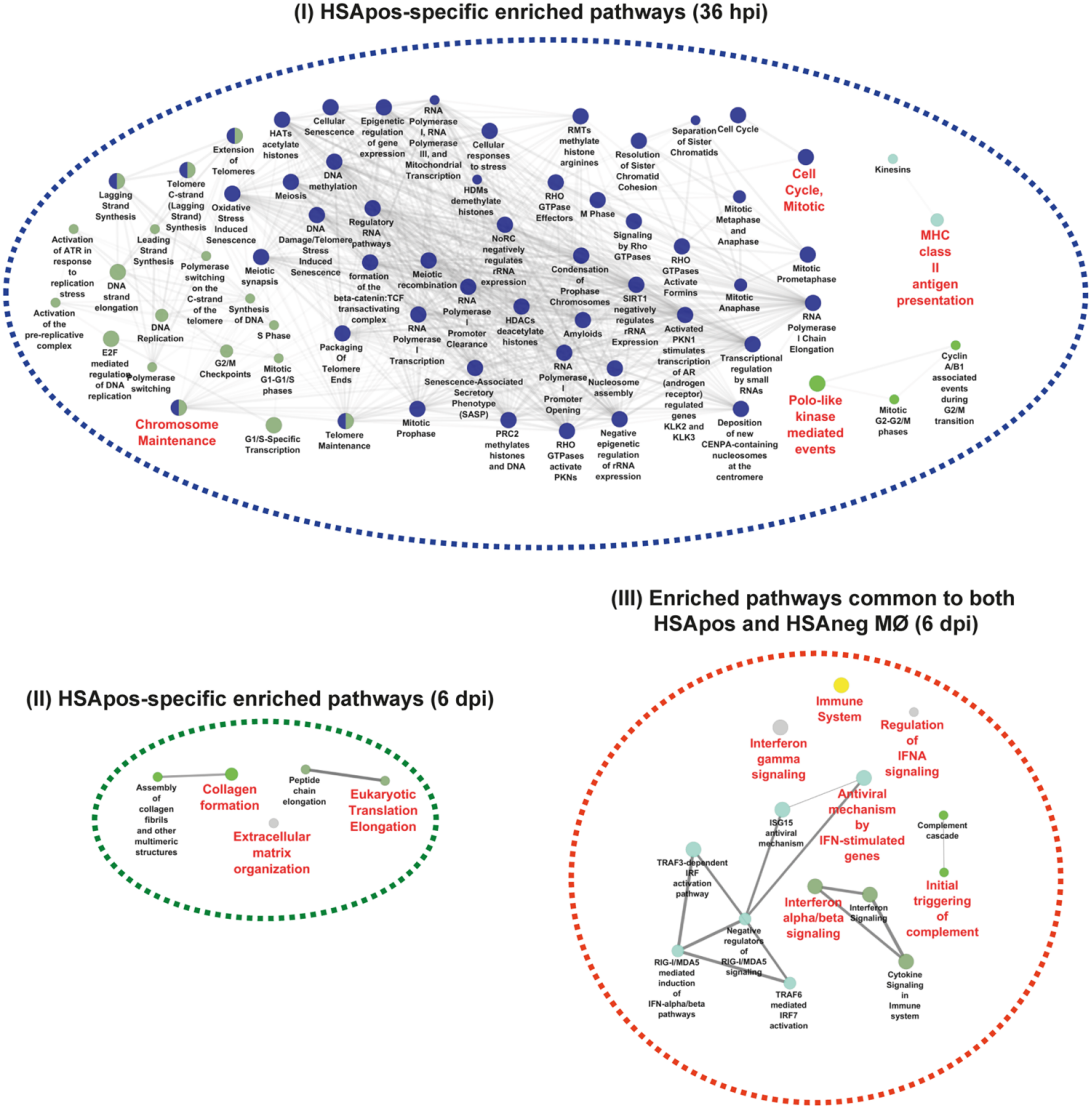
HIV-1 productive infection induces a global remodeling in the MØ transcriptome. Gene ontology analysis of differentially expressed transcripts shows a specific enrichment of DNA-associated pathways in HIV-1-infected MØ at 36 hpi (panel I). An enrichment of ECM- and translation-associated pathways is seen in HSApos MØ at 6 dpi (panel II). IFN-related genes are commonly expressed in HSApos and HSAneg MØ populations at 6 dpi (panel III). The complete list of enriched pathways are displayed in the Supplementary Table S4.

Next, we performed a single cluster analysis and comparison of clusters with the gene ontology tool called ClueGO to define the enriched biological pathways. Using the lists of DEGs identified in all populations by RNAseq at 36 hpi, we found the following statistically over-represented categories in HSApos MØ: cell cycle, telomere maintenance, polo-like kinase-mediated signaling events and presentation of the MHC class II antigen (Fig. 3, panel I). Surprisingly, very few gene ontology (GO) categories were found to be specifically over-represented in HSApos MØ at 6 dpi. They were mostly associated with extracellular matrix (ECM), remodeling and translation elongation (Fig. 3, panel II). Numerous genes associated with over-represented GO categories in HSApos cells at 6 dpi were also detected in HSAneg MØ such as key members of the TRAF3/6-mediated IFN-regulatory factor (IRF) activation or type-I IFN-induced antiviral restriction mechanisms (Fig. 3, panel III). Similar statistically over-represented categories were found when comparing the transcriptomic profiles independently for each cell population at 36 hpi and 6 dpi (Supplementary Fig. S2 and Supplementary Tables S3 and S4).

**Figure 4:**
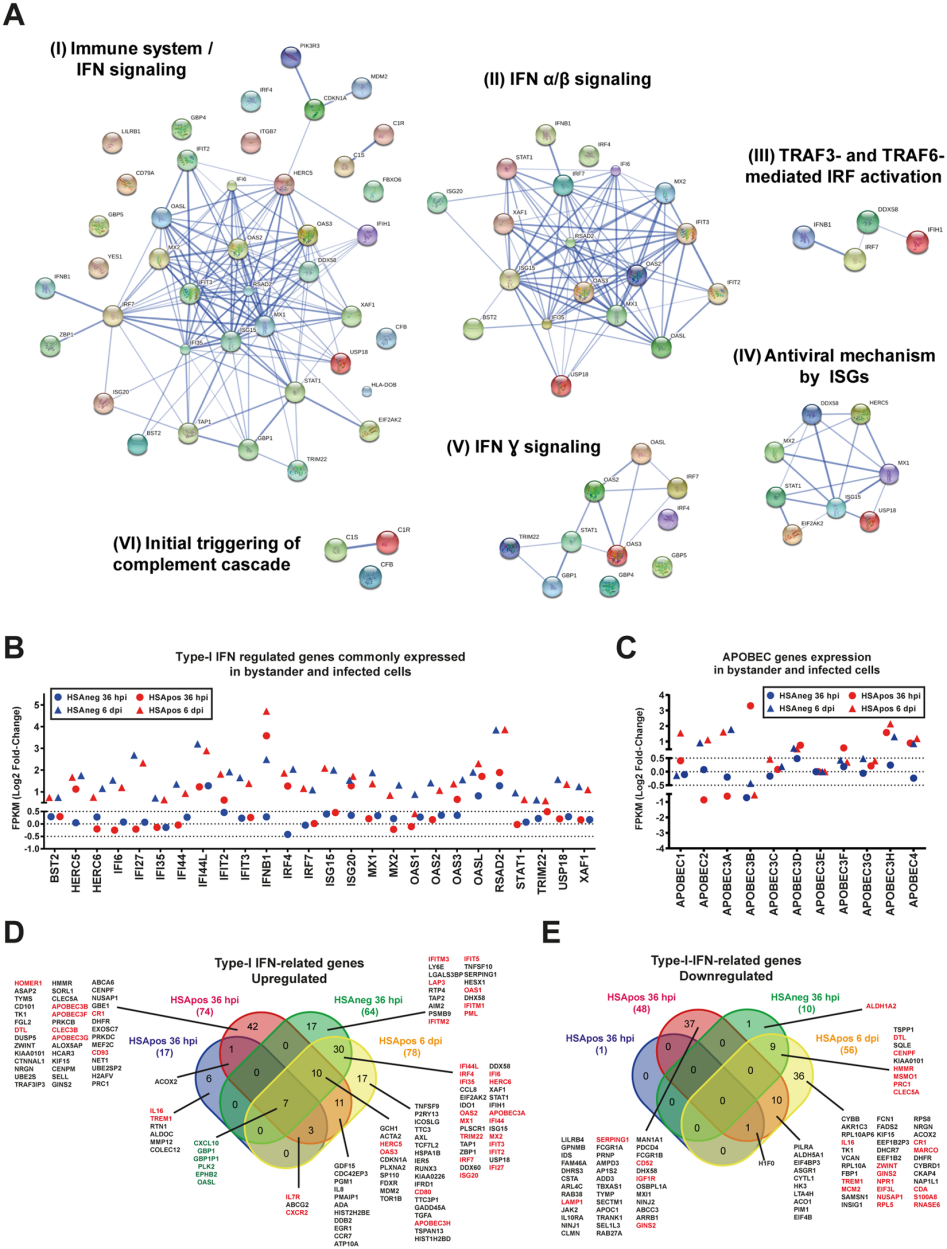
Type-I IFN response is detected in both HSAneg and HSApos MØ. (**A**) Commonly enriched transcripts in HSAneg and HSApos MØ at 6 dpi are associated with type-I (IFNα and IFNβ) and type-II (IFNγ) IFN signaling pathways. (**B**) Transcriptional expression of type-I IFN-associated genes is commonly modulated in HSApos and HSAneg MØ. Data are presented as Log2 FC of FPKM. (**C**) Transcriptional expression of several members of the APOBEC family genes is affected in both HSAneg and HSApos MØ. Expression of most type-I IFN-associated transcripts is significantly up-regulated in both HSAneg and HSApos MØ (**D**) but a large number of these genes is also down-regulated (**E**) (Cut-off: FC = 1.5, *p* value = 0.001). A complete list displaying the distribution of DEGs is shown in the Supplementary Table S12.

We next interrogated the STRING (Search Tool for the Retrieval of Interacting Genes/Proteins) biological database to identify known and predicted protein-protein interactions in the studied MØ populations. We found a significant enrichment of IFN-stimulated genes (ISGs), particularly for type-I ISGs associated to IFNα and IFNβ signaling (Fig. 4A, panels I, II and III). Expression of these genes was correlated with the modulation of the transcription factor IRF7, which is affected by IFNβ1 (Fig. 4A, panel IV). Interestingly, expression of the IFNβ1 mRNA encoding for this protein was specifically increased in HSApos cells at 6 dpi (data not shown). IRF7 regulation was also linked to IFNγ signaling together with IRF4 and STAT1 transcription factors in association with the oligo-adenylate synthases OAS2, OAS3 and OASL and the guanylate binding proteins GBP1, GBP4 and GBP5 (Fig. 4A, panel V). Finally, we detected also a cluster of ISGs associated with an initial triggering of the complement cascade like CS1, C1R and CFB (Fig. 4A, panel VI). Although no particular modulation of most ISGs could be detected for both HSAneg and HSApos MØ at 36 hpi, a significant increase was seen at 6 dpi. Surprisingly, after only 36 hpi, the level of expression for HERC5, ISG20 and RSAD2 antiviral genes was increased only in HSApos MØ. Other ISGs such as IFIT2, IRF4, IFI44L, OAS3 and OASL were found to follow a similar pattern of expression (Fig. 4B). Despite these common patterns seen in both MØ populations, a large subset of 132 type-I IFN-related genes, such as members of the APOBEC family (APOBEC1, APOBEC3B and APOBEC3F), was still over-expressed in HSApos cells (Fig. 4C). In contrast, since the total number of genes modulated in bystanders was globally more limited compared to HIV-1-infected cells (Fig. 2), the cluster of ISGs specifically augmented in this population was consequently smaller with only 24 specific DEGs. Interestingly, previously described resistance genes such as OAS1 or members of the IFITM family (IFITM1, IFITM2 and IFITM3) were over-represented in this particular cluster (Fig. 4D). Expression levels for all transcripts from the IFI, IFITM, TRIM, IRF and TLR gene families are shown in the Supplementary Fig. S3. Transcriptional expression of *interferon-inducible* (*IFI*) and *interferon inducible trans-membrane* (*IFITM*) genes is associated with different patterns in HSAneg and HSApos MØ. For example, IFI6, IFI27, IFI35, IFI44, IFIH1, IFIT1B, IFIT3 and IFITM3 are specifically enriched at 6 dpi in both HSAneg and HSApos populations compared to mock-infected cells (see Supplementary Fig. S3A). An enhanced expression of IFI27L1 is seen in HSApos MØ at 36 hpi, whereas ITM4P and IFITM10 are enriched only at 6 dpi in the same MØ population. IFIT5 and IFITM2 transcripts are increased in HSAneg MØ at 6 dpi. Finally, expression of IFI44L is augmented in both HSAneg and HSApos MØ compared to mock-infected samples. Transcriptional expression of most tripartite motif (TRIM) proteins was not significantly modulated in the different samples at 36 hpi and 6 dpi (see Supplementary Fig. S3B). Only TRIM15 is specifically enriched at 6 dpi in both HSAneg and HSApos MØ with a significant diminution in HSApos MØ at 36 hpi. TRIML1, TRIM9, TRIM40 and TRIM55 expression is also enriched in these two populations at 6 dpi but a significant increase can be observed in HSApos MØ at 36 hpi. Transcriptional expression of the IFN-regulated factors IRF4 and IRF7 is increased at 6 dpi for both HSAneg and HSApos populations compared to mock-infected cells (see Supplementary Fig. S3C). IRF4 is also augmented at 36 hpi in HSApos cells but not in HSAneg MØ. In contrast, IRF5 and IRF6 are both diminished in HSApos MØ at 36 hpi. Members of the Toll-like receptor (TLR) family display different patterns of expression within HSAneg and HSApos populations (see Supplementary Fig. S3D). We also analyzed the pattern of expression of ISGs that were reduced at the transcriptional level. Figure 4E shows that in contrast to up-regulated transcripts, the large majority of genes which expression was significantly decreased were modulated only in the HSApos population.

**Figure 5:**
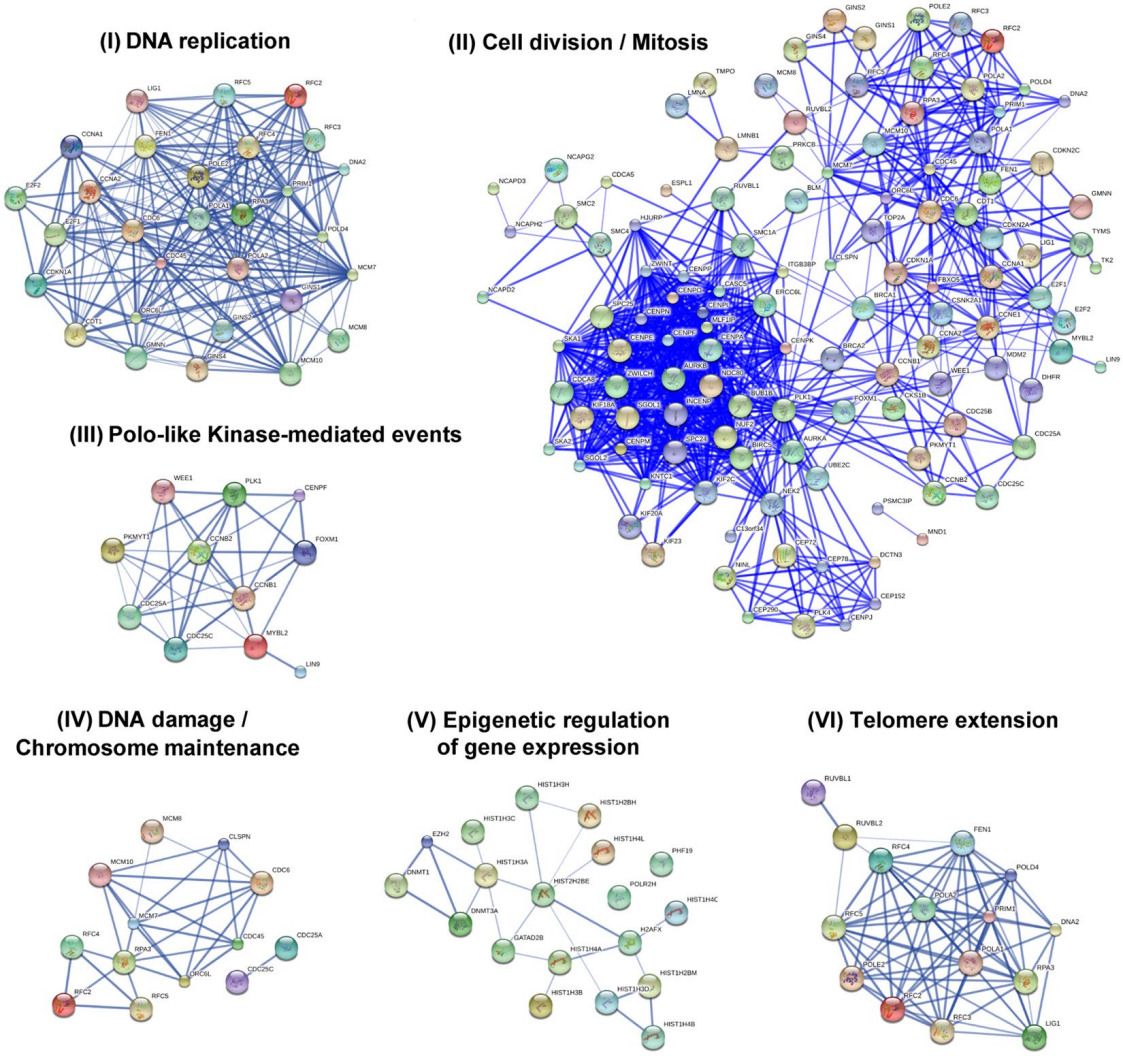
HIV-1 replication induces a rapid modulation of genes involved in DNA- and chromatin-related events. Complex DNA- and chromatin-associated signaling networks are strongly modulated in HSApos MØ at 36 hpi for the following enriched GO-terms: DNA replication (**I**), cell division and mitosis (**II**), Polo-like kinase-mediated events (**III**), DNA damage and chromosome maintenance (**IV**), epigenetic regulation of gene expression (**V**) and telomere extension (**VI**).

To decipher molecular interactions over-represented in HSApos MØ at 36 hpi, we performed a STRING Network and Pathway analysis of the different Reactome clusters. Importantly, we found that productive HIV-1 infection is associated with an early activation of DNA replication components such as DNA polymerase subunits (POLA1, POLA2, POLD4 and POLE2), the origin replication complex protein ORC6L, the DNA primase PRIM1, the flap endonuclease FEN1, the DNA helicase and the DNA ligase LIG1 (Fig. 5, panel I). Proteins encoded by these genes associate into highly dynamic complexes progressing along the DNA replication fork to separate and replicate the two DNA strands. Their activation is tightly regulated by expression of other genes that were also enriched in HSApos MØ. For example, the geminin GMNN, the replication protein RPA3, the chromatin licensing and DNA replication factor CDT1, proteins of the GINS family (GINS1, GINS2 and GINS4), the mini-chromosome maintenance factors (MCM7, MCM8 and MCM10), the CDCA/CDK1 complex and activators of the replication factor C family (RFC2, RFC3, RFC4 and RFC5) are all upstream regulators of DNA replication factors that are transcriptionally up-regulated in the HSApos population. Interestingly, a network of more than 60 genes corresponding to factors associated with the cell cycle was also significantly enriched in HSApos MØ at 36 hpi (Fig. 5, panel II). Furthermore, genes involved in PLK1- (Polo-Like Kinase) and WEE1-mediated signaling were also significantly enriched in HSApos MØ (Fig. 5, panel III). Phosphorylation events mediated by these two proteins control the activity of cyclins (CCN) as well as cyclin-dependent phosphatases (CDC) and kinases (CDK).

**Figure 6:**
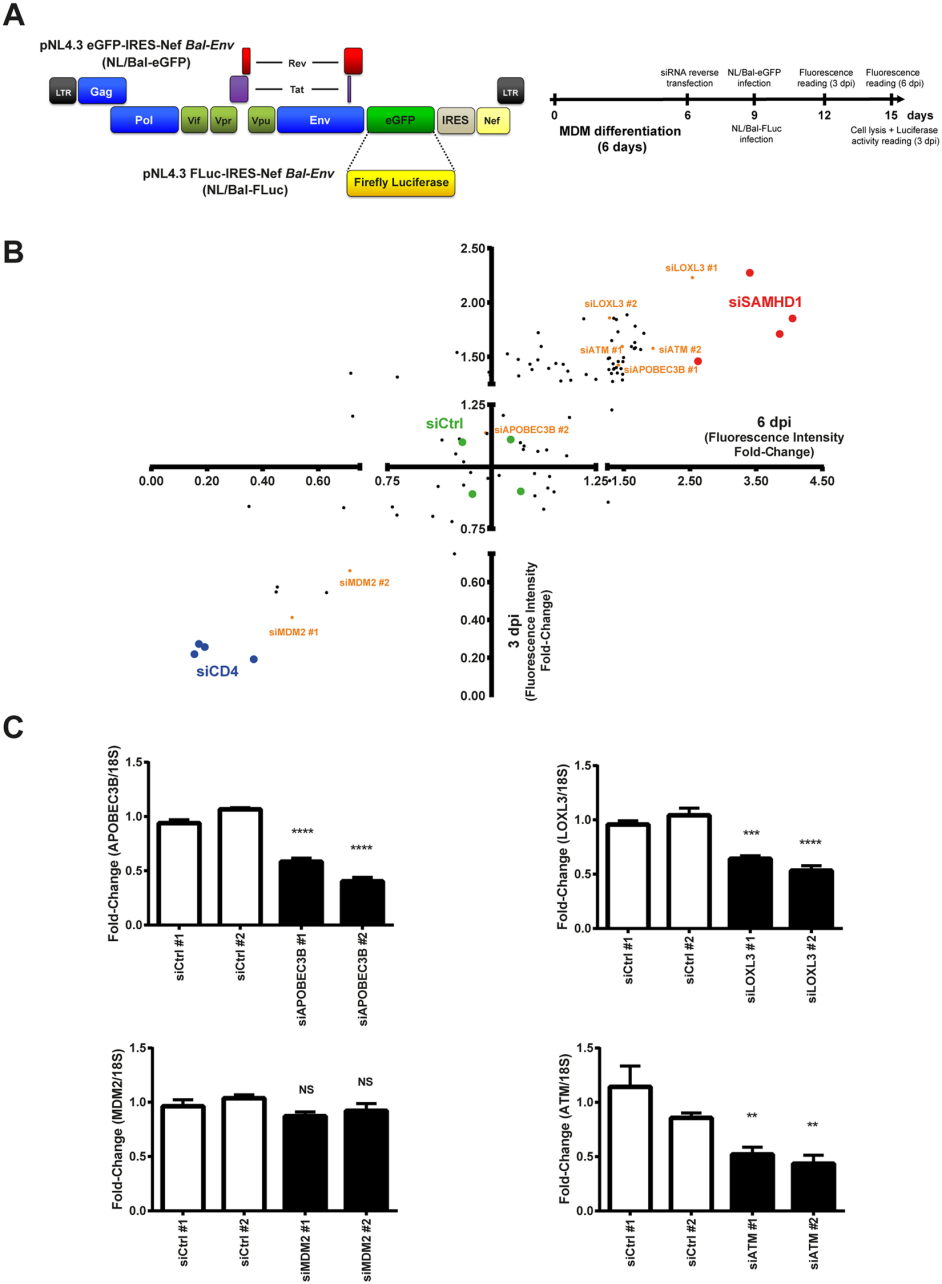
siRNA screen reveals new regulators of HIV-1 replication in MØ. (**A**) Vector maps of fully competent infectious molecular clones NL/Bal-eGFP and NL/Bal-FLuc (left panel) and schematic representation of the experimental protocol used in the siRNA screen (right panel). (**B**) siRNA sequences directed against fifty different host genes selected from RNAseq analyses allowed identification of interesting candidates for new regulators of HIV-1 replication in MØ (*orange dots*). siRNAs directed against SAMHD1 were used as controls for restriction factors (*red dots*) and siRNAs against CD4 were used as controls for susceptibility factors (*blue dots*). Data are presented as the mean of six independent experiments. (**C**) Transcriptional expression of *MDM2*, *LOXL3*, *ATM* and *APOBEC3B* genes was monitored following siRNA delivery. Data are presented as the mean ± SD. Asterisks denote statistically significant data (**p < 0.01; ***p < 0.001; ****p < 0.0001; NS: not significant).

Proteins of the mini-chromosome maintenance (MCM proteins) mediate reorganization of histones to ensure the transcription of viral genes in dividing cells. MCM7 expression was significantly enhanced in HSApos MØ (Fig. 5, panel IV), thus suggesting that this gene could act as a major regulator for HIV-1 post-integration DNA repair in MØ. Expression levels for all members of the histone family that have been detected by RNAseq are displayed in the Supplementary Fig. S4 (panels A to E). In the group of 71 histone genes, which transcription could be detected by RNAseq, 45 of them were significantly up-regulated in HSApos MØ when compared to mock-infected cells (Log2 FC > 0.5). Moreover, a network involving the histone isoforms HIST1H3A, HIST1H4A and HIST2H2BE, together with the transcriptional repressor GATAD2B appears to be associated with histone signaling (Fig. 5, panel V). An up-regulation of the DNA methylases DNMT1 and DNMT3A was associated with the transcription of histones in HSApos MØ (Fig. 6, panel V). No significant modulation was observed for the transcription of histone methyl transferases (HMTs) but expression of the histone acetylase HAT1 was slightly increased in HSApos cells at 36 hpi. The histone deacetylase HDAC9 was also augmented whereas HDAC11 expression was significantly decreased (see Supplementary Tables S1 and S2). Finally, the expression of several genes associated with extension and maintenance of telomeres was modulated in the HSApos population. This network involves the AAA-ATPases RUVBL1 and RUVBL2 together with the DNA replication complex PRIM1/POLA/POLD/POLE/FEN1/DNA2/LIG1 (Fig. 5, panel VI).

Additional comparative analyses revealed that HSAneg MØ are weakly affected by HIV-1 at 36 hpi in contrast to HSApos cells, which display a large number of enriched signaling pathways essentially associated with DNA and chromatin remodeling. However, at 6 dpi, the number of enriched signaling pathways is markedly enhanced in HSAneg MØ and present common features with the HSApos cell population (Figs 3 and 4A and Supplementary Tables S5 and S6). To clarify further differences between HIV-1-directed effects in HSApos MØ and indirect effects in both HSApos and HSAneg cell populations, we carried out additional comparative analyses of transcription factors and cytokine/receptor signaling pathways that were differentially regulated in the studied MØ populations. We found that the IRF1- and IRF2-regulated genes were enriched in HSApos MØ at both 36 hpi and 6 dpi but only at 6 dpi in the HSAneg cell population. Moreover, IRF7-regulated genes were enriched in both HSApos and HSAneg MØ but only at 6 dpi (Fig. S5A and Supplementary Table S7). Genes regulated by IFN-γ were significantly increased in both HSApos and HSAneg MØ at 6 dpi (Fig. S5B and Supplementary Table S8). Given that IFN-γ-mediated signaling induces MØ polarization, we compared our data in light of the previously published transcriptomic profiles of MØ displaying M1 and M2 phenotype^15^. There is a HIV-1-mediated modulation of genes normally associated with the M2 phenotype in all experimental conditions tested. However, several M1-related genes were modulated in both HSApos and HSA-neg MØ at 6 dpi (Fig. S5C to F and Supplementary Table S9).

### siRNA screening reveals some cell-encoded determinants regulating HIV-1 replication in MØ

To identify certain host factors modulating HIV-1 biology in MØ, we selected fifty different genes showing a differential expression at the mRNA level and tested two different siRNA sequences per target. To monitor the level of HIV-1 replication in MØ with a high-throughput reading, we engineered two reporter virus constructs containing the complete HIV-1 genome transcriptionally fused either to the *enhanced green fluorescent protein* (NL/Bal-eGFP) or *firefly luciferase* gene (NL/Bal-FLuc) (Fig. 6, left panel). MØ were first transfected with siRNAs and inoculated with HIV-1 before monitoring the virus-encoded reporter signal (Fig. 6, right panel). Negative controls consisted of siRNAs specific for nonsense/scrambled sequences (green dots), whereas siRNAs directed against SAMHD1 (red dots) and CD4 (blue dots) mRNAs were used as positive controls for viral restriction and susceptibility factors, respectively (Fig. 6B). Validations of knockdown efficiency for these controls are presented in the Supplementary Fig. S6A. Gene silencing of MDM2 was found to have an overall negative impact on HIV-1 replication in MØ (Fig. 6B, lower left). Cell viability was not affected by siRNAs targeting MDM2 (Fig. S6B), thus indicating that the lower level of viral replication was associated with a reduced expression of the targeted transcripts. Surprisingly, knockdown of most genes associated with type-I IFN signaling (i.e. MX1, MX2, TRIM22, OAS3, OASL, ISG15 and CXCL11) had a minimal efficiency to modulate HIV-1 replication (data not shown). However, siRNAs specific for the lysyl oxidase-like 3 (LOXL3), apolipoprotein APOBEC3B and DNA damage-associated Ser/Thr kinase ATM induced an increase in virus-dependent reporter activity (Fig. 6B, upper right). In particular, knockdown of LOXL3 led to a significant augmentation in reporter gene activity almost comparable to the one obtained after SAMHD1 silencing. These results were confirmed using different kinetics of siRNA transfection and with a NL/Bal-FLuc molecular clone to exclude any bias linked to eGFP expression or to a change in cell auto-fluorescence (data not shown). Knockdown efficiency of the tested siRNAs was validated by quantitative real-time polymerase chain reaction (qRT-PCR) assays (Fig. 6C). Indeed, the relative mRNA expression of all transcripts targeted was efficiently decreased by their specific siRNA sequences after 72 h of siRNA transfection, except for MDM2. However, expression of MDM2 transcripts was reduced after 24 and 48 h of siRNA transfection (data not shown).

## Discussion

To study the direct and indirect HIV-1-mediated molecular signatures in MØ, we developed an *in vitro* experimental model system allowing separation and enrichment of MØ productively infected with HIV-1 (HSApos) and bystanders (HSAneg) from an initial heterogeneous population (Fig. 1). This approach has allowed us to identify host genes in human MØ, which are modulated upon productive infection with HIV-1. Our experiments were performed with human peripheral blood monocytes that were differentiated into uncommitted/nonpolarized MØ (M0 phenotype). This information is of prime importance since the activation status of human MØ has been reported to affect HIV-1 replication^16^. Our technical procedure is based on replication-competent HIV-1 particles coding for the murine HSA cell surface molecule. It is conceivable that engagement of the reporter murine HSA membrane glycoprotein during the purification procedure might alter expression of some host genes. However, the HSA-mediated signaling could be significantly reduced given that most of the physiological murine interaction partners are lacking in human MØ. Importantly, the immunomagnetic bead-based experimental procedure that we used to isolate and purify HSA-expressing human MØ was performed at 4 °C. In signal transduction experiments, negative controls consisted of cell that were left at 4 °C because there are minimal signaling events at this temperature. Nevertheless, it is anticipated that silencing of host genes induced possibly by the murine HSA reporter molecule would not affect HIV-1 replication in human MØ upon siRNA-mediated gene silencing.

We used next-generation sequencing (RNAseq) analyses to compare the transcriptomic profiles of these two MØ populations. Our work revealed that productively infected and bystander MØ exhibit not only several common but also specific features. For example, remodeling of DNA and chromatin appears to be essentially associated with early productive HIV-1 infection (36 hpi), whereas activation of IFN-related antiviral immunity was seen in both cell populations at a later time point (6 dpi) (Fig. 2). Moreover, we report that productively infected MØ underwent a much more extensive alteration of their transcriptome compared to bystanders at the earliest time point studied (36 hpi). These results are consistent with microarray data previously described in primary human CD4^+^ T cells^17^. Although virus replication is repressed by ISGs^18^, modulation of several ISGs was not specific to HSApos MØ since it was also seen in HSAneg cells (Figs 3 to 5). Surprisingly, expression of genes from the APOBEC family was found primarily in HSApos MØ (Fig. 4C), whereas members of the IFITM family were more significantly modulated in HSAneg cells (Fig. 4D). Viral spliced mRNA transcripts were not found in bystanders, thus suggesting that the molecular environment in these cells might not be favorable for the early steps of HIV-1 life cycle. The IFITM proteins have been recently identified for their role in resistance to HIV-1 infection^19^. A specific expression of IFITM1, IFITM2 and IFITM3 in HSAneg MØ could therefore suggest that these ISGs are major determinants of HIV-1 restriction in this cell population. In contrast, up-regulation of APOBEC3A, APOBEC3B, APOBEC3F and APOBEC3G transcription in HSApos MØ did not appear to be sufficient *per se* to limit viral replication (Fig. 4C). Our observations demonstrate that the kinetic of changes in the transcriptome profiles is directly influenced by the temporal dynamics of HIV-1 life cycle in human MØ. Indeed, at an early time point following infection (36 hpi), remodeling of host DNA and chromatin is essential to achieve an efficient integration and transcription of the HIV-1 genome. Thereafter, when virus infection is productive (i.e. several days post-infection), activation of the type-I IFN-related antiviral immunity is taking place. However, bystanders can also be affected by other means. For example, HIV-1-infected cells can produce some soluble factors that can engage cell surface receptors in bystanders and generate an amplification loop through IRF (e.g. IRF7), a process leading to induction of IFN-regulated genes. Interestingly, we report here that some of these transcriptional changes are associated with expression of several genes linked to M1 polarization (Figs 4 and S5).

Since the transcriptome of bystanders was weakly affected early after virus infection (36 hpi), most DEGs identified were specific to HSApos cells (Figs 3 and 5). A detailed analysis of these genes revealed a significant enrichment of DNA replication- and chromatin remodeling-associated pathways required for successful HIV-1 infection in human MØ (Fig. 5 and see Supplementary Fig. S4). The delicate balance between HIV-1 latency and productive infection is known to be controlled by epigenetic regulation of gene expression, which is itself tightly regulated by DNA methylation and modifications of amino acid residues contained in the histone tails^20^. Here we show that the expression of DNA methylases DNMT1 and DNMT3A was increased in HSApos MØ together with the histone acetyl transferase HAT1 and histone deacetylase HDAC9. In contrast, expression of HDAC11 was lower in such cells. A similar pattern of gene expression was found for the nuclear transporter and transcriptional repressor GATAD2B, a major regulator for the induction of the nucleosome remodeling and histone deacetylation complex (NuRD) containing HDAC1 and HDAC2^21^. In contrast with other retroviruses, HIV-1 does not require cell division or breakdown of the nuclear envelope for the nuclear import of its genome. MØ are highly differentiated and are generally considered as non-dividing cells. However, it has been reported that MØ possess self-renewal capacities *in vivo* ^6^. Interestingly, HIV-1 can increase survival of MØ by promoting telomerase activity and can thus make them as long-term viral sanctuaries^7^.

We found that the transcriptional changes triggered by HIV-1 in MØ appear to be specifically balanced between responses that limit or promote viral replication. In bystanders, an activation of the type-I IFN signaling is initiated in the same way as in productively infected MØ, thus suggesting that these two populations are exposed to the same triggering signals. In contrast, induction of susceptibility genes linked to DNA reorganization required for viral integration and transcription appeared to be specific to MØ productively infected with HIV-1 (Fig. 5). Among the fifty tested DEGs in our siRNA screening experiments, some of them were found to modulate HIV-1 replication. MDM2 was the only host gene apart from CD4 that can facilitate virus replication, even if no mRNA knockdown was detected at the time of virus delivery (72 h post-transfection). Nevertheless, expression of the MDM2 transcript was altered at 24 and 48 h after siRNA transfection, indicating a rapid turnover for this gene. Therefore, the MDM2-mediated inhibitory effect on HIV-1 might be associated to other host factors which regulation depends on MDM2. Additional studies are needed to solve this issue.

We also identified several DEGs that can restrict HIV-1 replication in human MØ. Among them, APOBEC3B, ATM and LOXL3 were the most interesting candidates. Interestingly, members of the APOBEC3 family have been shown to cooperate with ATM serine/threonine kinase to repair double-stranded DNA breaks (DSB) in the context of HIV-1-infected cells containing deaminated viral DNA^22^. Of note, LOXL3 knockdown induced an up-regulation of virus gene expression almost as efficient as the one triggered by silencing SAMHD1 (Fig. 6). Interestingly, LOXL3 mRNA expression was significantly decreased in HIV-1-infected MØ compared to uninfected and bystander cells, implying that lower levels of this transcript are correlated with a molecular environment more favorable for HIV-1 replication. Inhibition of LOXL3 activity using siRNA silencing led to a noticeable increase in HIV-1 mRNA and protein expression in MØ. Proteins of the Lysyl Oxidase (LOX) family can affect protein behaviors in different ways such as proteasomal degradation, subcellular localization, structural organization, or biochemical activity^23^. Moreover, LOX activity is known to be responsible for post-translational modifications of the histones H1, H2 and H3, which are associated with a negative regulation of gene transcription^24, 25^. Finally, it has been reported that expression of a recombinant propeptide displaying LOX activity interferes with the DSB repair response in cancer cells by abrogating ATM phosphorylation^26^. Studies are currently underway to identify the exact mechanism by which LOXL3 can limit HIV-1 replication in human MØ.

## Methods

### Ethics statement, cell culture and viral infection

All experimental protocols described in the present study were approved by the Bioethics Committee from the Centre Hospitalier Universitaire de Québec-Université Laval. The methods were performed in accordance with the Institutional guidelines and regulations. All participants provided a written informed consent. Human monocytes from healthy donors were differentiated in MØ by adherence for 3 days in the presence of RPMI-1640 culture medium supplemented with M-CSF (25 ng/mL) and 10% human AB serum. Cells were maintained in culture medium in absence of M-CSF for an additional 3 days, treated with Accutase™ (Stem Cell Technologies) for 60 to 90 min, and detached with a soft cell scraper (Sarstedt). Cells were plated at 2.5 × 10^5^ cells/mL for 24 (cell-culture coated dishes) or 72 h (Ultra-Low attachment dishes). The purity and identity of human monocyte-derived MØ used in our work were assessed by flow cytometric analyses. In brief, cells were labeled with monoclonal antibodies specific for the pan-macrophage marker CD68, T-cell marker CD3 and B-cell marker CD19. Given that CD68 is primarily localized in lysosomes and endosomes, cells were first permeabilized before staining. The majority of human monocyte-derived MØ expressed CD68 (greater than 97%) while most cells were negative for CD3 and CD19 (less than 1%) (see Supplementary Fig. S7). MØ were infected with replication-competent NL/Bal-HSA, NL/Bal-eGFP or NL/Bal-FLuc with multiplicities of infection (MOI) ranging from 0.004 to 2.5. In each experiment, a minimum of three different donors was processed.

### Products

RPMI-1640 and DMEM cell culture media as well as fetal bovine serum were provided by Thermo Fisher Scientific (Nepean, ON). Human AB serum was purchased from Valley Biomedical (Winchester, VA) and Penicillin/Streptomycin 100x solution from Thermo Fisher Scientific. Accutase™ cell detachment solution, monoclonal antibodies (mAbs) and other products used for flow cytometry and immunofluorescence are described in the supplemental experimental procedures.

### Flow cytometry and cell sorting studies

Cells were plated in hydrophobic-coated dishes (Sarstedt) or Ultra-Low attachment dishes (Corning) and detached by pipetting up and down after 30 min of incubation in PBS/5 mM EDTA/1% BSA. Next, HSApos and HSAneg MØ were isolated using biotinylated anti-HSA (clone M1/69; eBiosciences) and anti-Biotin Magnetic Beads (Miltenyi) in a MS separation column (Miltenyi). Cells were blocked with PBS/20% Normal Goat Serum (NGS)/10% human AB serum/1% BSA/5 mM EDTA and next labelled with PE-conjugated anti-HSA, APC-conjugated anti-HSA, APC-conjugated anti-CD4 (eBiosciences), or FITC-conjugated anti-p24 mAb (clone KC57, Beckman Coulter).

### siRNA transfection

We diluted 0.35 μl Lipofectamine RNAi-Max (Life Technologies) into 25 μl of RPMI-1640 medium into each well. We next diluted 2.5 pmol of each individual siRNA tested into 25 μl of RPMI-1640 medium into each well, and incubated for 20 min at room temperature. We next added 50 μl of diluted cell suspension mixture containing 5 × 10^4^ MØ on top of the complex to obtain a final concentration of 20 nM siRNA. Cells were placed at 37 °C in the presence of 5% CO_2_ for 2 h before adding 100 μl of RPMI-1640 medium supplemented with 20% human AB serum (10% final concentration). After 72 h, the medium was replaced and cells were infected with NL/Bal-eGFP or NL/Bal-FLuc for 3 to 6 additional days before measuring reporter gene activity (72 hpi and 6 dpi, respectively). The complete list of siRNAs used for transfection can be found as Supplementary Table S10.

### RNA-sequencing analyses

We performed virus infection assays using MØ from 20 different donors and RNAseq studies were carried out with 4 representative samples. Purified MØ populations (HSAneg and HSApos) were resuspended and frozen in QIAzol lysis buffer. Total RNA was isolated from sorted cell populations using the miRNeasy Kit from QIAGEN (Valencia, CA). RNA-seq libraries were prepared using the Scriptseq V2 complete gold kit (Epicentre) and the TrueSeq RNA Library Prep Kit V2 (Illumina) following manufacturer’s instructions. RNA integrity was assessed using RNA 6000 nano and pico kits. DNA libraries were quantified using high sensitivity DNA kits following manufacturer’s instructions (Agilent). Details for the bioinformatics analysis are described in the supplemental experimental procedures.

### qRT-PCR

Total RNA was isolated with QIAGEN miRNeasy kits and reverse transcribed into complementary DNA (cDNA) with a M-MLV RT Polymerase (Promega). Oligonucleotides used for the specific amplification of target genes are described in the supplemental experimental procedures. qRT-PCR measurements were performed with Fast SYBR Green PCR master mix in a two-step Real-Time PCR System (Applied Biosystems). Amplification of target genes was normalized to 18S ribosomal RNA and expressed as relative expression using the comparative change in cycle threshold (Delta Delta cT method). All primers used for qRT-PCR analyses are described in the Supplementary Table S11.

### Data availability statement

The datasets generated during and/or analyzed during the current work are available from the corresponding author on reasonable request.

## Electronic supplementary material

Supplementary Information

Table S1

Table S2

Table S3

Table S4

Table S5

Table S6

Table S7

Table S8

Table S9

Table S10

Table S11

Table S12
