# Supplementary material for: Global Mapping of the Macrophage-HIV-1 Transcriptome Reveals that Productive Infection Induces Remodeling of Host Cell DNA and Chromatin
Source: Sci Rep. 2017 Jul 12;7:5238. doi: 10.1038/s41598-017-05566-9 (PMC5507862; doi:10.1038/s41598-017-05566-9)
Supplement: Supplementary file 1 — Supplementary Information [file 41598_2017_5566_MOESM1_ESM.pdf]

Global Mapping of the Macrophage-HIV-1 Transcriptome Reveals that Productive Infection Induces Remodeling of Host Cell DNA and Chromatin

Alexandre Deshiere[a], Charles Joly-Beauparlant[a], Yann Breton[a], Michel Ouellet[a], Frédéric Raymond[a], Robert Lodge[b], Corinne Barat[a], Marc-André Roy[a], Jacques Corbeil[a,c], and Michel J. Tremblay[a, d,#]

[a]Axe des Maladies Infectieuses et Immunitaires, Centre de Recherche du Centre Hospitalier Universitaire de Québec-Université Laval, Québec, Canada; [b]Institut de Recherches Cliniques de Montréal, Montréal, Québec, Canada; [c]Département de médecine moléculaire, Faculté de médecine, Université Laval, Québec, Canada; [d]Département de microbiologie-infectiologie et immunologie, Faculté de médecine, Université Laval, Québec, Canada

Running title: HIV-1 affects host DNA and chromatin in macrophages

#Address correspondence to Michel J. Tremblay, michel.j.tremblay@crchudequebec.ulaval.ca

Supplementary Experimental Procedures

Molecular constructs. NL/Bal-eGFP and NL/Bal-FLuc were engineered from NLENG1-IRES[1] (a kind gift from David N. Levy), in which a portion of the wild-type X4 envelope from NL4.3 was replaced with a corresponding fragment of the Bal R5 envelope from pNL4.3 *Bal env*[2].

Detachment of MØ for flow cytometry analysis and cell sorting. To overcome the constraint of sorting heavily adherent cells such as human MØ, Ultra Low Attachment dishes (ULA, Corning®) were used. These dishes are coated with a hydrophobic gel, which limits cell-surface interactions. This approach allows a complete differentiation and attachment of MØ in the presence of M-CSF and strongly facilitates cell detachment when using buffers supplemented with EDTA. Using these experimental conditions, cell viability was close to 90-100% after cell detachment (10 min PBS - 5 mM EDTA) whereas cell culture coated or non-treated polystyrene dishes required >30 min incubation with trypsin-EDTA and usually lead to more than 50% mortality (unpublished observations). Non-specific binding sites were blocked using a 20 min incubation period with PBS supplemented with 0.5% BSA, 20% normal goat serum, 10% human AB serum and 5 mM EDTA. Abs used for flow cytometry and cell sorting studies were tested at a 1:100 final concentration and purchased from eBioscience (San Diego, CA), except for anti-p24 (clone KC57) that was obtained from Beckman Coulter (Mississauga, ON). Anti-SAMHD1 mAb was purchased from Cell Signaling Technology (Danvers, MA).

Virus production and infection of MØ. Virus stocks were prepared by transient transfection of human embryonic kidney 293T cells using the calcium phosphate precipitation technique, as previously published[3]. Multiplicity of infection (MOI) of our virus preparations was determined by calculating the $TCID_{50}$ using the Spearman-Karber method following infection of the

indicator cell line TZM-Bl[4]. MØ were infected with the NL/Bal-HSA reporter virus at a MOI of 0.1 for immunomagnetic sorting experiments. Controls consisting of mock-infected MØ were obtained by using cell-free supernatants from 293T cells transiently transfected with an empty pCDNA3.1 vector. A MOI of 0.5 was used for studies with NL/eBal-GFP and NL/Bal-Fluc reporter viruses. For all experiments, the virus was diluted in culture medium supplemented with 10% human AB serum. HIV-1 infection was maintained for 72 h without changing the culture medium.

Viability Assays. Viability of MØ after siRNA transfection was assessed using CellTiter-Blue Cell Viability and CellTiter 96 AQueous Non-Radioactive Cell Proliferation Assays (MTS) following manufacturer's instructions (Promega, Madison, WI).

Western blot analysis. MØ were reverse transfected with siRNAs either specific for nonsense/scrambled sequences (used as negative controls) or SAMHD1 and next plated at 2 x 10^5 cells per well in 24 well plates. MØ were lysed for 15 min at 4°C in 150 µL per well of T8 lysis buffer (Urea 7 M, Thiourea 2 M, 3-[(3-cholamidopropyl)dimethylammonio]-1-propanesulfonate (CHAPS) 3 % (w/v), dithiothreitol (DTT) 20 mM, and 5 mM tris(2-carboxyethyl)phosphine (TCEP) containing 1% (v/v) of the protease inhibitor solution P8340 (Sigma Aldrich, Saint-Louis, MO). Protein concentrations of MØ lysates were determined by a Bradford assay using Quick Start™ Bradford 1x Dye Reagent (Bio-Rad, Mississauga, ON). Total lysates from MØ were frozen at -80°C until used. MØ lysates (20 µg per lane) were separated on a 10% SDS-PAGE and transferred on a 0.45 µm PVDF membrane (Thermo Fisher Scientific). Molecular weight markers (RPN800E; GE Healthcare, Mississauga, ON) were also loaded to determine the molecular weight of target proteins. Protein expression was monitored by standard immunoblotting techniques using primary Abs directed against HIV-1 p24 protein

(clone 183-H12-5C, 1:1000; cat#1513; NIH AIDS Reagent program) and actin (goat anti-hActin, 1:2000, clone I-19; Santa Cruz Biotechnology; Dallas, TX) as a loading control. Non-specific binding sites of the PVDF membrane were first blocked in PBS supplemented with 0.1% (v/v) Tween-20 (PBST) and 5% BSA (Fraction V Standard Grade Bovine Serum Albumin, Cohn Fraction V from Fitzgerald Industries International, Acton, MA) for 1 hr at room temperature before being incubated with primary Abs overnight at 4°C in blocking buffer. Membranes were then washed three times in PBST and incubated for 1h with HRP-conjugated secondary Abs (rat anti-mouse IgG from Rockland Immunochemicals, Limerick, PA; donkey anti-goat IgG from Santa Cruz Biotechnology) diluted at 1:10 000 in PBST. Membranes were again washed three times in PBST before adding ECL substrate (Western Lightning Plus-ECL; Perkin Elmer) and exposed on film (Universal X-ray, Pointe-Claire, QC). Films were developed and scanned. Minimal brightness and contrast processing of the scanned image were performed using Adobe Photoshop CS5 and the figure was generated using Adobe Illustrator CS5.

RNA-sequencing analyses. The Trimmomatic v0.30 software was used to trim fastq sequences and remove adapters using an ILLUMINACLIP of 2:30:15, a TRAILING of 30 and a MINLEN of 32 (A.M. Bolger, M. Lohse, and B. Usadel, Bioinformatics 30:2114-2120, 2014). The reads were aligned on the hg19 genome using Tophat v2.0.8b using fr-unstranded library-type and using gene annotations downloaded from UCSC on March 9, 2012 (through Illumina iGenomes). Duplicates were marked and reads were sorted using the MarkDuplicates and SortSam algorithms from Picard tools v1.88. Raw counts were obtained with htseq-count v0.5.4p1 and FPKM were calculated using Cufflinks v2.0.2 with a max-bundle-frags value of 1000000000 and afr-unstranded library-type [5]. Differential gene expression was computed with the EdgeR package v3.4.2 with normalization to data from mock-infected cells as described previously[6].

Enrichment of each cell population and the specificity of the associated sequencing reads were assessed by an alignment to the HIV-1 genome. The coverage was evaluated with the human genome (Hg19) alignment. A total of 21800 different human transcripts could be detected in MØ with coverage of 100 million reads per sample. Among them, 18013 transcripts were corresponding to protein-coding mRNA sequences and we could compare the expression of 11220 transcripts in the different conditions after applying a transcript detection threshold (TDT) limit of 0.1 fragment per kilobase per million of RNAseq reads.

Gene ontology and pathway networks analysis. Lists of genes were selected applying a cut-off of 1.5 for Fold-Change and $p < 0.001$ for p values. To decipher functionally grouped gene ontology and pathway annotation networks, functional enrichment analysis was performed using ClueGO, a cytoscape plugin. The statistical test used Reactome 05.05.2015[7,8] for the enrichment analysis was a right-sided hypergeometric test with a Benjamini-Hochberg correction and a kappa score of 0.4, GO Term grouping (Pathway/Network connectivity), and a medium network specificity (Homo sapien, 3 minimum genes, 3 % genes; pV < 0.05)[9,10]. Signaling networks were obtained from STRING 9.1 with experiments active prediction methods and a confidence score of 0.400 (medium)[11,12]. Analyses of transcription factor regulation enrichments were performed with the Database for Annotation, Visualization and Integrated Discovery (DAVID 6.8)[13,14] using the Protein Interaction tool (UCSC TFBS, Homo Sapiens). For receptor-associated signaling pathways, we used EnrichNet Network-based enrichment analysis[15,16], applying the NCI-Nature Pathway Interaction and STRING networks databases.

Statistical analysis. Means of raw data were compared using the two-way ANOVA with Sidak's multiple comparison tests. *P* values lower than 0.05 were deemed statistically significant.

Calculations were performed with the GraphPad Prism version 6.02 for Windows (GraphPad Software, La Jolla, CA).

Supplementary Figure Legends

Figure S1. Kinetics of NL/Bal-HSA replication in MØ and purity of HSAneg and HSApos fractions isolated by immunomagnetic sorting. (A) MØ from three distinct donors were inoculated with increasing amounts of NL/Bal-HSA (MOI ranging from 0.004 to 2.5) and virus-mediated reporter gene activity was assessed at 6 dpi. Data are presented as the mean of triplicate samples ± SD. (B) MØ from three different donors were infected with NL/Bal-HSA (MOI: 1) and virus replication was measured at the indicated time points. Data are presented as the mean of triplicate samples ± SD. (C) MØ were infected with NL/Bal-HSA before isolation of HSAneg and HSApos populations by immunomagnetic sorting at 36 and 72 hpi. Finally, immunocaptured cells were subjected to western blotting analyses using anti-p24 (clone 183-H12-5C) and anti-actin (clone I-19) mAbs. The results shown are representative from 1 out of 3 independent experiments. (D) MØ were infected with NL/Bal-HSA before isolation of HSAneg and HSApos populations by immunomagnetic sorting at 24, 72 hpi and 6 dpi. Mock-infected MØ were used as negative controls. Next, levels of Tat mRNA and 18S rRNA were measured by qRT-PCR analyses. Data are presented as the mean of triplicate samples ± SD. The results shown are representative from 1 out of 3 independent experiments.

Figure S2. Comparative analyses of DEGs in HSAneg and HSApos MØ at 36 hpi and 6 dpi. A Venn diagram analysis of Differentially Expressed Genes (DEGs) at 36 hpi and 6dpi shows a time-dependent regulation of host gene transcription in HSAneg and HSApos MØ. The listed numbers indicate the total number of genes in each area. Diagrams were generated at http://bioinformatics.psb.ugent.be/webtools/Venn/. A complete list displaying the distribution of of DEGs is depicted in the Supplementary Table S3, whereas the corresponding enriched pathways are presented in the Supplementary Table S4.

Figure S3. Type-I IFN-related response is seen in both HSAneg and HSApos cell fractions. MØ were infected with NL/Bal-HSA before isolation of HSAneg and HSApos populations using an immunomagnetic sorting approach at 36 hpi and 6 dpi. Mock-infected MØ were used as negative controls. Next, RNAseq analyses were performed in studied cell samples. Data are presented as Log2 FC of FPKM. Transcriptional expression of numerous genes linked with the type-I IFN-associated response was monitored and compared in both HSAneg and HSApos MØ (panel A: *interferon-inducible* (*IFI*), *interferon-induced proteins with tetracopeptide repeats* (IFIT) and *interferon inducible trans-membrane* (*IFITM*) genes; panel B: *tripartite motif* (TRIM) genes; panel C: *IFN-regulated factor* (IRF) genes; and panel D: *Toll-like receptor* (TLR) genes.

Figure S4. HIV-1 induces chromatin reorganization early after infection. MØ were infected with NL/Bal-HSA before isolation of HSAneg and HSApos populations by immunomagnetic sorting at 36 hpi and 6 dpi. Mock-infected MØ were used as negative controls. Next, RNAseq analyses were performed in studied cell samples. Data are presented as Log2 FC of FPKM. Transcriptional expression of several histone family members was assessed and compared in both HSAneg and HSApos MØ.

Figure S5. HIV-1 induces type-I IFN signaling in both HSAneg and HSApos MØ. (A) Comparative analysis of DEGs in both HSAneg and HSApos MØ using Database for Annotation, Visualization and Integrated Discovery (DAVID) indicate that regulation of genes associated with Interferon Regulatory Factors (IRF) transcriptional activity occurs in both MØ populations at 6 dpi. (B) A similar analysis for cytokine/receptor-associated pathways using EnrichNet shows that IFN-γ downstream signaling events are modulated in both HSAneg and HSApos MØ at 6 dpi. Detailed lists of enriched transcription factors and cytokine/receptor-associated downstream signaling pathways are listed in Supplementary Tables S7 and S8,

respectively. (C) Comparative analyses between DEGs in HSAneg MØ and lists of genes previously described to be modulated in MØ treated with IFN-γ and LPS (M1 phenotype) or IL-4/IL-13 (M2 phenotype)[17-19] shows that no particular polarization occurs in these cells at 36 hpi. Interestingly, 23 M1-associated DEGs were found to be transcriptionally modulated in HSApos MØ at 36 hpi. (D) Similar analyses demonstrate that a large panel of M1-associated DEGs (i.e. 42) linked to type-I IFN signaling is regulated in HSAneg cells without induction of M2-associated DEGs at 6 dpi. A comparable profile of M1-associated DEGs is detected in HSApos MØ at 6 dpi with no particular modulation of M2-associated DEGs. A complete list of the repartition of DEGS is presented in the Supplementary Table S9. All Venn diagrams were generated at http://bioinformatics.psb.ugent.be/webtools/Venn/. The numbers shown indicate the total numbers in each area.

Figure S6. siRNA-based screening to search for host factors modulating HIV-1 replication in MØ. (A) Transcriptional expression of SAMHD1 (left panel) and CD4 (right panel) is reduced when using sequence-specific siRNAs. Data are presented as the mean ± SD of triplicate samples. Asterisks denote statistically significant data (***$p < 0.001$; ****$p < 0.0001$). (B) Analysis of MØ viability after siRNA transfection shows that this experimental approach induces minimal cell toxicity. Indeed, both CellTiter-Blue cell viability assay and CellTiter 96 AQueous Non-Radioactive cell proliferation Assay (MTS) experiments show homogeneous distribution of the different transfected siRNA conditions. Data are presented as the mean ± SD of three independent experiments.

Figure S7. Flow cytometry analysis of human monocyte-derived MØ used in our work. Human monocyte-derived MØ from 4 distinct healthy donors were labeled first with monoclonal antibodies specific for the pan-macrophage marker CD68 (as suggested by the Referee), T-cell

marker CD3, and B-cell marker CD19. Next, cells were blocked with 20% NGS/10% human AB serum/1% BSA/5mM EDTA and labelled with conjugated secondary antibodies before flow cytometry analyses.

References

1 Levy, D., Aldrovandi, G., Kutsch, O. & Shaw, G. Dynamics of HIV-1 recombination in its natural target cells. *Proceedings of the National Academy of Sciences of the United States of America* **101**, 4204 (2004).

2 Dornadula, G., Zhang, H., Shetty, S. & Pomerantz, R. HIV-1 virions produced from replicating peripheral blood lymphocytes are more infectious than those from nonproliferating macrophages due to higher levels of intravirion reverse transcripts: implications for pathogenesis and transmission. *Virology* **253**, 10 (1999).

3 Cantin, R., Fortin, J. & Tremblay, M. The amount of host HLA-DR proteins acquired by HIV-1 is virus strain-and cell type-specific. *Virology* **218**, 372 (1996).

4 Platt, E. J., Wehrly, K., Kuhmann, S. E., Chesebro, B. & Kabat, D. Effects of CCR5 and CD4 Cell Surface Concentrations on Infections by Macrophagetropic Isolates of Human Immunodeficiency Virus Type 1. *Journal of Virology* **72**, 2855 (1998).

5 Trapnell, C. *et al.* Differential gene and transcript expression analysis of RNA-seq experiments with TopHat and Cufflinks. *Nature protocols* **7**, 562-578 (2012).

6 Robinson, M. D., McCarthy, D. J. & Smyth, G. K. edgeR: a Bioconductor package for differential expression analysis of digital gene expression data. *Bioinformatics* **26**, 139 (2010).

7 Fabregat, A. *et al.* The Reactome pathway Knowledgebase. *Nucleic Acids Res* **44**, D481-487, doi:10.1093/nar/gkv1351 (2016).

8 Croft, D. *et al.* The Reactome pathway knowledgebase. *Nucleic Acids Res* **42**, D472-477, doi:10.1093/nar/gkt1102 (2014).

9 Bindea, G. *et al.* ClueGO: a Cytoscape plug-in to decipher functionally grouped gene ontology and pathway annotation networks. *Bioinformatics* **25**, 1091 (2009).

10 Shannon, P. *et al.* Cytoscape: A Software Environment for Integrated Models of Biomolecular Interaction Networks. *Genome Research* **13**, 2498 (2003).

11 Snel, B., Lehmann, G., Bork, P. & Huynen, M. STRING: a web-server to retrieve and display the repeatedly occurring neighbourhood of a gene. *Nucleic Acids Research* **28**, 3442 (2000).

12 Franceschini, A. *et al.* STRING v9. 1: protein-protein interaction networks, with increased coverage and integration. *Nucleic Acids Research* **41**, D808 (2013).

13 Huang, D. W. *et al.* The DAVID Gene Functional Classification Tool: a novel biological module-centric algorithm to functionally analyze large gene lists. *Genome Biol* **8**, R183, doi:10.1186/gb-2007-8-9-r183 (2007).

14 Huang, D. W. *et al.* DAVID Bioinformatics Resources: expanded annotation database and novel algorithms to better extract biology from large gene lists. *Nucleic Acids Res* **35**, W169-175, doi:10.1093/nar/gkm415 (2007).

15 Glaab, E., Baudot, A., Krasnogor, N., Schneider, R. & Valencia, A. EnrichNet: network-based gene set enrichment analysis. *Bioinformatics* **28**, i451-i457, doi:10.1093/bioinformatics/bts389 (2012).

16 Schaefer, C. F. *et al.* PID: the Pathway Interaction Database. *Nucleic Acids Res* **37**, D674-679, doi:10.1093/nar/gkn653 (2009).

17 Cassol, E., Cassetta, L., Alfano, M. & Poli, G. Macrophage polarization and HIV-1 infection. *Journal of leukocyte biology* **87**, 599 (2010).

18 Becker, M. *et al.* Integrated Transcriptomics Establish Macrophage Polarization Signatures and have Potential Applications for Clinical Health and Disease. *Sci Rep* **5**, 13351, doi:10.1038/srep13351 (2015).

19 Martinez, F. O., Gordon, S., Locati, M. & Mantovani, A. Transcriptional profiling of the human monocyte-to-macrophage differentiation and polarization: new molecules and patterns of gene expression. *J Immunol* **177**, 7303-7311 (2006).

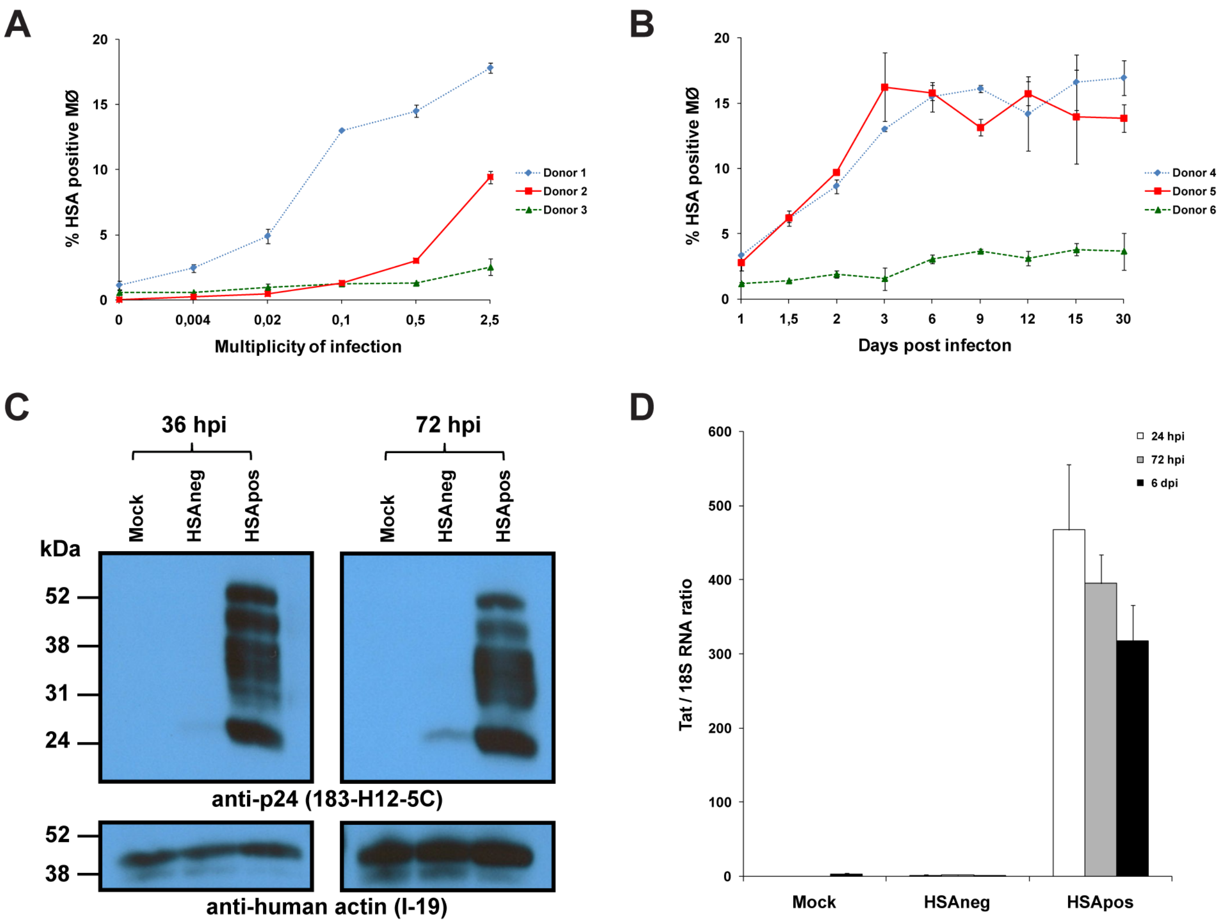

A
20
15
10
5
0
% HSA positive MØ
0
0,004
0,02
0,1
0,5
2,5
Multiplicity of infection
Donor 1
Donor 2
Donor 3
B
20
15
10
5
0
% HSA positive MØ
1
1,5
2
3
6
9
12
15
30
Days post infecton
Donor 4
Donor 5
Donor 6
C
36 hpi
72 hpi
Mock
HSAneg
HSApos
Mock
HSAneg
HSApos
kDa
52
38
31
24
anti-p24 (183-H12-5C)
52
38
anti-human actin (I-19)
D
600
500
400
300
200
100
0
Tat / 18S RNA ratio
24 hpi
72 hpi
6 dpi
Mock
HSAneg
HSApos

Figure S1

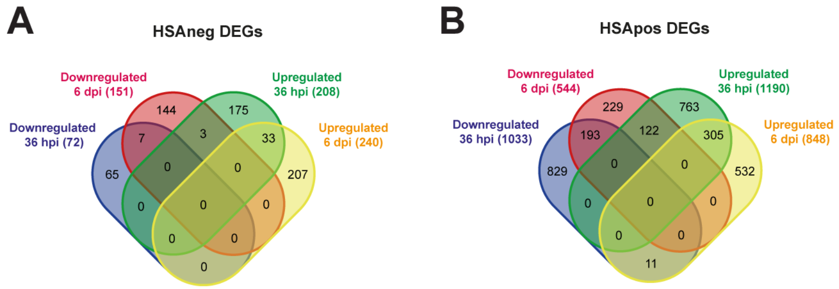
A
HSAneg DEGs
Downregulated
6 dpi (151)
Upregulated
36 hpi (208)
Downregulated
36 hpi (72)
Upregulated
6 dpi (240)
144
175
7
3
33
65
0
0
207
0
0
0
0
0
0
0
B
HSApos DEGs
Downregulated
6 dpi (544)
Upregulated
36 hpi (1190)
Downregulated
36 hpi (1033)
Upregulated
6 dpi (848)
229
763
193
122
305
829
0
0
532
0
0
0
0
0
0
11

Figure S2

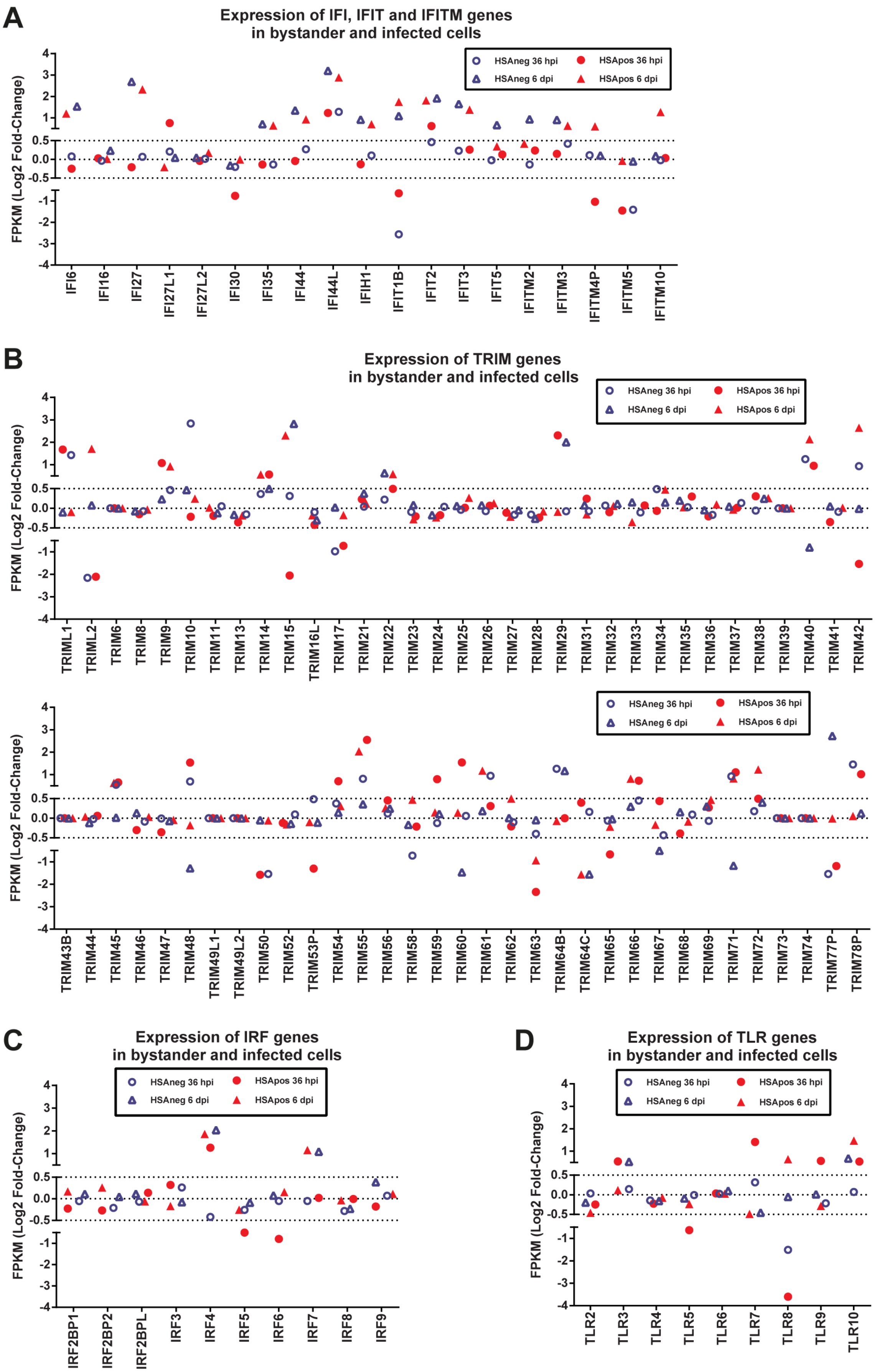
A
Expression of IFI, IFIT and IFITM genes
in bystander and infected cells
HSAneg 36 hpi
HSApos 36 hpi
HSAneg 6 dpi
HSApos 6 dpi
FPKM (Log2 Fold-Change)
4
3
2
1
0.5
0.0
-0.5
-1
-2
-3
-4
IFI6
IFI16
IFI27
IFI27L1
IFI27L2
IFI30
IFI35
IFI44
IFI44L
IFIH1
IFIT1B
IFIT2
IFIT3
IFIT5
IFITM2
IFITM3
IFITM4P
IFITM5
IFITM10
B
Expression of TRIM genes
in bystander and infected cells
HSAneg 36 hpi
HSApos 36 hpi
HSAneg 6 dpi
HSApos 6 dpi
FPKM (Log2 Fold-Change)
TRIML1
TRIML2
TRIM6
TRIM8
TRIM9
TRIM10
TRIM11
TRIM13
TRIM14
TRIM15
TRIM16L
TRIM17
TRIM21
TRIM22
TRIM23
TRIM24
TRIM25
TRIM26
TRIM27
TRIM28
TRIM29
TRIM31
TRIM32
TRIM33
TRIM34
TRIM35
TRIM36
TRIM37
TRIM38
TRIM39
TRIM40
TRIM41
TRIM42
HSAneg 36 hpi
HSApos 36 hpi
HSAneg 6 dpi
HSApos 6 dpi
FPKM (Log2 Fold-Change)
TRIM43B
TRIM44
TRIM45
TRIM46
TRIM47
TRIM48
TRIM49L1
TRIM49L2
TRIM50
TRIM52
TRIM53P
TRIM54
TRIM55
TRIM56
TRIM58
TRIM59
TRIM60
TRIM61
TRIM62
TRIM63
TRIM64B
TRIM64C
TRIM65
TRIM66
TRIM67
TRIM68
TRIM69
TRIM71
TRIM72
TRIM73
TRIM74
TRIM77P
TRIM78P
C
Expression of IRF genes
in bystander and infected cells
HSAneg 36 hpi
HSApos 36 hpi
HSAneg 6 dpi
HSApos 6 dpi
FPKM (Log2 Fold-Change)
IRF2BP1
IRF2BP2
IRF2BPL
IRF3
IRF4
IRF5
IRF6
IRF7
IRF8
IRF9
D
Expression of TLR genes
in bystander and infected cells
HSAneg 36 hpi
HSApos 36 hpi
HSAneg 6 dpi
HSApos 6 dpi
FPKM (Log2 Fold-Change)
TLR2
TLR3
TLR4
TLR5
TLR6
TLR7
TLR8
TLR9
TLR10

Figure S3

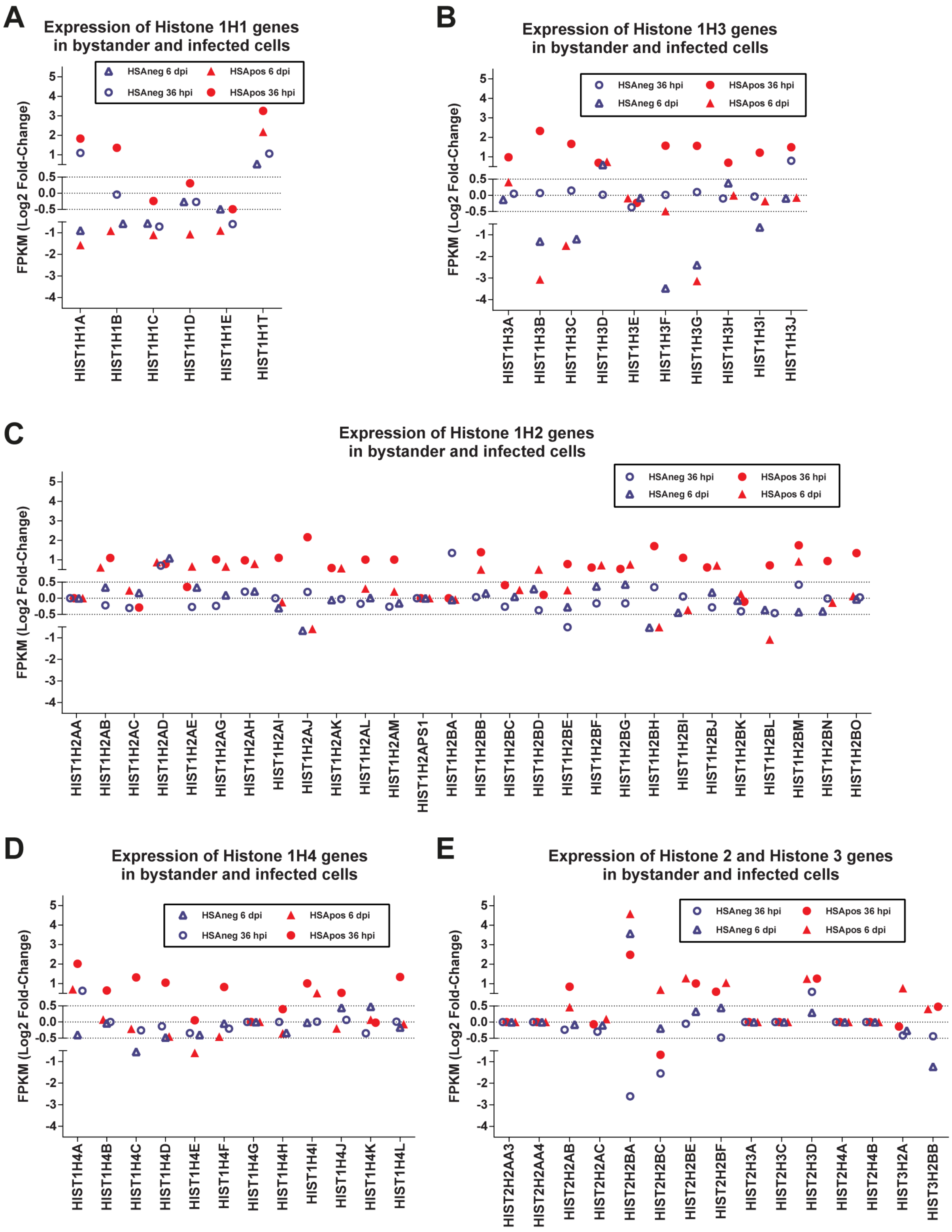
A
Expression of Histone 1H1 genes in bystander and infected cells
HSAneg 6 dpi
HSApos 6 dpi
HSAneg 36 hpi
HSApos 36 hpi
FPKM (Log2 Fold-Change)
HIST1H1A
HIST1H1B
HIST1H1C
HIST1H1D
HIST1H1E
HIST1H1T
B
Expression of Histone 1H3 genes in bystander and infected cells
HIST1H3A
HIST1H3B
HIST1H3C
HIST1H3D
HIST1H3E
HIST1H3F
HIST1H3G
HIST1H3H
HIST1H3I
HIST1H3J
C
Expression of Histone 1H2 genes in bystander and infected cells
HIST1H2AA
HIST1H2AB
HIST1H2AC
HIST1H2AD
HIST1H2AE
HIST1H2AG
HIST1H2AH
HIST1H2AI
HIST1H2AJ
HIST1H2AK
HIST1H2AL
HIST1H2AM
HIST1H2APS1
HIST1H2BA
HIST1H2BB
HIST1H2BC
HIST1H2BD
HIST1H2BE
HIST1H2BF
HIST1H2BG
HIST1H2BH
HIST1H2BI
HIST1H2BJ
HIST1H2BK
HIST1H2BL
HIST1H2BM
HIST1H2BN
HIST1H2BO
D
Expression of Histone 1H4 genes in bystander and infected cells
HIST1H4A
HIST1H4B
HIST1H4C
HIST1H4D
HIST1H4E
HIST1H4F
HIST1H4G
HIST1H4H
HIST1H4I
HIST1H4J
HIST1H4K
HIST1H4L
E
Expression of Histone 2 and Histone 3 genes in bystander and infected cells
HIST2H2AA3
HIST2H2AA4
HIST2H2AB
HIST2H2AC
HIST2H2BA
HIST2H2BC
HIST2H2BE
HIST2H2BF
HIST2H3A
HIST2H3C
HIST2H3D
HIST2H4A
HIST2H4B
HIST3H2A
HIST3H2BB

Figure S4

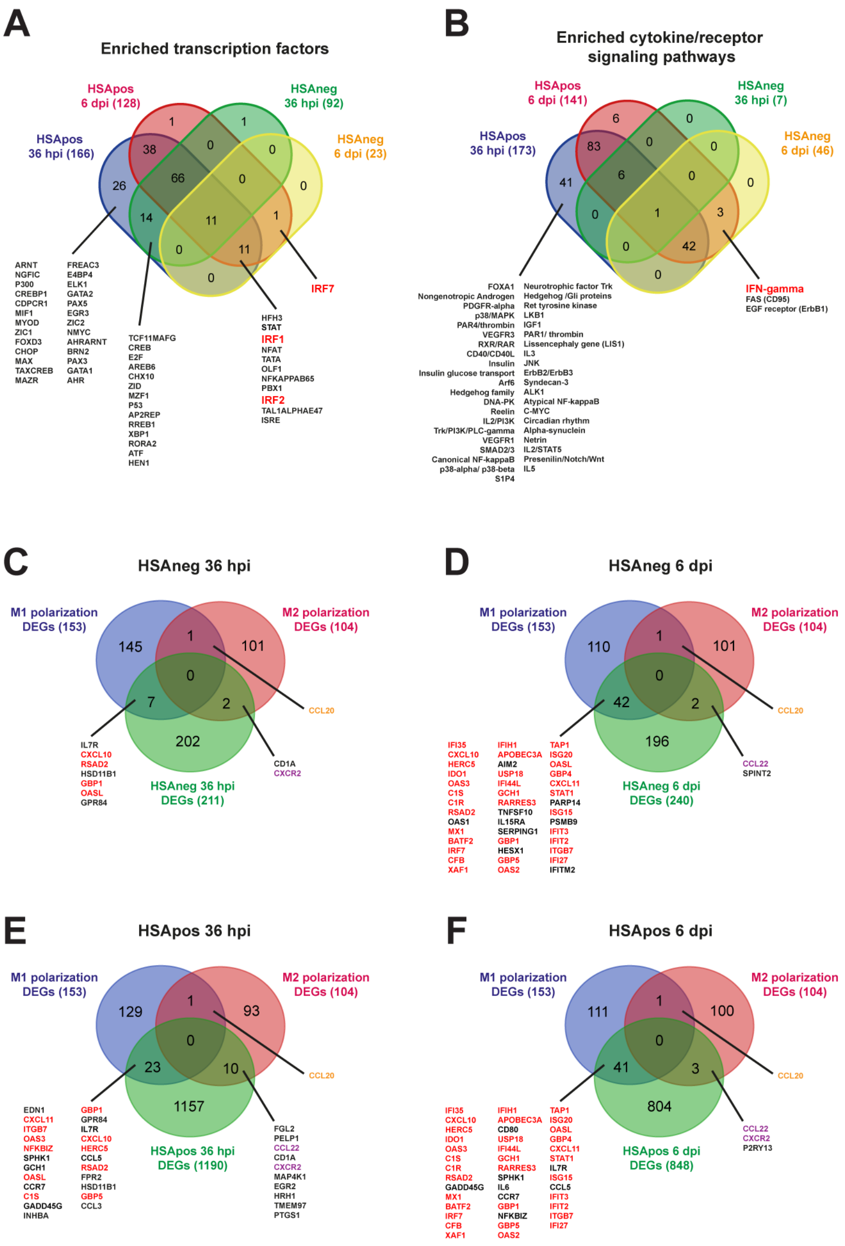
A
Enriched transcription factors
HSApos 6 dpi (128)
HSAneg 36 hpi (92)
HSApos 36 hpi (166)
HSAneg 6 dpi (23)
1
1
38
0
0
26
66
0
0
14
11
1
0
11
0
ARNT
NGFIC
P300
CREBP1
CDPCR1
MIF1
MYOD
ZIC1
FOXD3
CHOP
MAX
TAXCREB
MAZR
FREAC3
E4BP4
ELK1
GATA2
PAX5
EGR3
ZIC2
NMYC
AHRARNT
BRN2
PAX3
GATA1
AHR
TCF11MAFG
CREB
E2F
AREB6
CHX10
ZID
MZF1
P53
AP2REP
RREB1
XBP1
RORA2
ATF
HEN1
HFH3
STAT
IRF1
NFAT
TATA
OLF1
NFKAPPAB65
PBX1
IRF2
TAL1ALPHAE47
ISRE
IRF7
B
Enriched cytokine/receptor signaling pathways
HSApos 6 dpi (141)
HSAneg 36 hpi (7)
HSApos 36 hpi (173)
HSAneg 6 dpi (46)
6
0
83
0
0
41
6
0
0
0
1
3
0
42
0
FOXA1
Nongenotropic Androgen
PDGFR-alpha
p38/MAPK
PAR4/thrombin
VEGFR3
RXR/RAR
CD40/CD40L
Insulin
Insulin glucose transport
Arf6
Hedgehog family
DNA-PK
Reelin
IL2/PI3K
Trk/PI3K/PLC-gamma
VEGFR1
SMAD2/3
Canonical NF-kappaB
p38-alpha/ p38-beta
S1P4
Neurotrophic factor Trk
Hedgehog /Gli proteins
Ret tyrosine kinase
LKB1
IGF1
PAR1/ thrombin
Lissencephaly gene (LIS1)
IL3
JNK
ErbB2/ErbB3
Syndecan-3
ALK1
Atypical NF-kappaB
C-MYC
Circadian rhythm
Alpha-synuclein
Netrin
IL2/STAT5
Presenilin/Notch/Wnt
IL5
IFN-gamma
FAS (CD95)
EGF receptor (ErbB1)
C
HSAneg 36 hpi
M1 polarization DEGs (153)
M2 polarization DEGs (104)
145
1
101
0
7
2
202
CCL20
IL7R
CXCL10
RSAD2
HSD11B1
GBP1
OASL
GPR84
CD1A
CXCR2
HSAneg 36 hpi DEGs (211)
D
HSAneg 6 dpi
M1 polarization DEGs (153)
M2 polarization DEGs (104)
110
1
101
0
42
2
196
CCL20
IFI35
CXCL10
HERC5
IDO1
OAS3
C1S
C1R
RSAD2
OAS1
MX1
BATF2
IRF7
CFB
XAF1
IFIH1
APOBEC3A
AIM2
USP18
IFI44L
GCH1
RARRES3
TNFSF10
IL15RA
SERPING1
GBP1
HESX1
GBP5
OAS2
TAP1
ISG20
OASL
GBP4
CXCL11
STAT1
PARP14
ISG15
PSMB9
IFIT3
IFIT2
ITGB7
IFI27
IFITM2
CCL22
SPINT2
HSAneg 6 dpi DEGs (240)
E
HSApos 36 hpi
M1 polarization DEGs (153)
M2 polarization DEGs (104)
129
1
93
0
23
10
1157
CCL20
EDN1
CXCL11
ITGB7
OAS3
NFKBIZ
SPHK1
GCH1
OASL
CCR7
C1S
GADD45G
INHBA
GBP1
GPR84
IL7R
CXCL10
HERC5
CCL5
RSAD2
FPR2
HSD11B1
GBP5
CCL3
HSApos 36 hpi DEGs (1190)
FGL2
PELP1
CCL22
CD1A
CXCR2
MAP4K1
EGR2
HRH1
TMEM97
PTGS1
F
HSApos 6 dpi
M1 polarization DEGs (153)
M2 polarization DEGs (104)
111
1
100
0
41
3
804
CCL20
IFI35
CXCL10
HERC5
IDO1
OAS3
C1S
C1R
RSAD2
GADD45G
MX1
BATF2
IRF7
CFB
XAF1
IFIH1
APOBEC3A
CD80
USP18
IFI44L
GCH1
RARRES3
SPHK1
IL6
CCR7
GBP1
NFKBIZ
GBP5
OAS2
TAP1
ISG20
OASL
GBP4
CXCL11
STAT1
IL7R
ISG15
CCL5
IFIT3
IFIT2
ITGB7
IFI27
HSApos 6 dpi DEGs (848)
CCL22
CXCR2
P2RY13

Figure S5

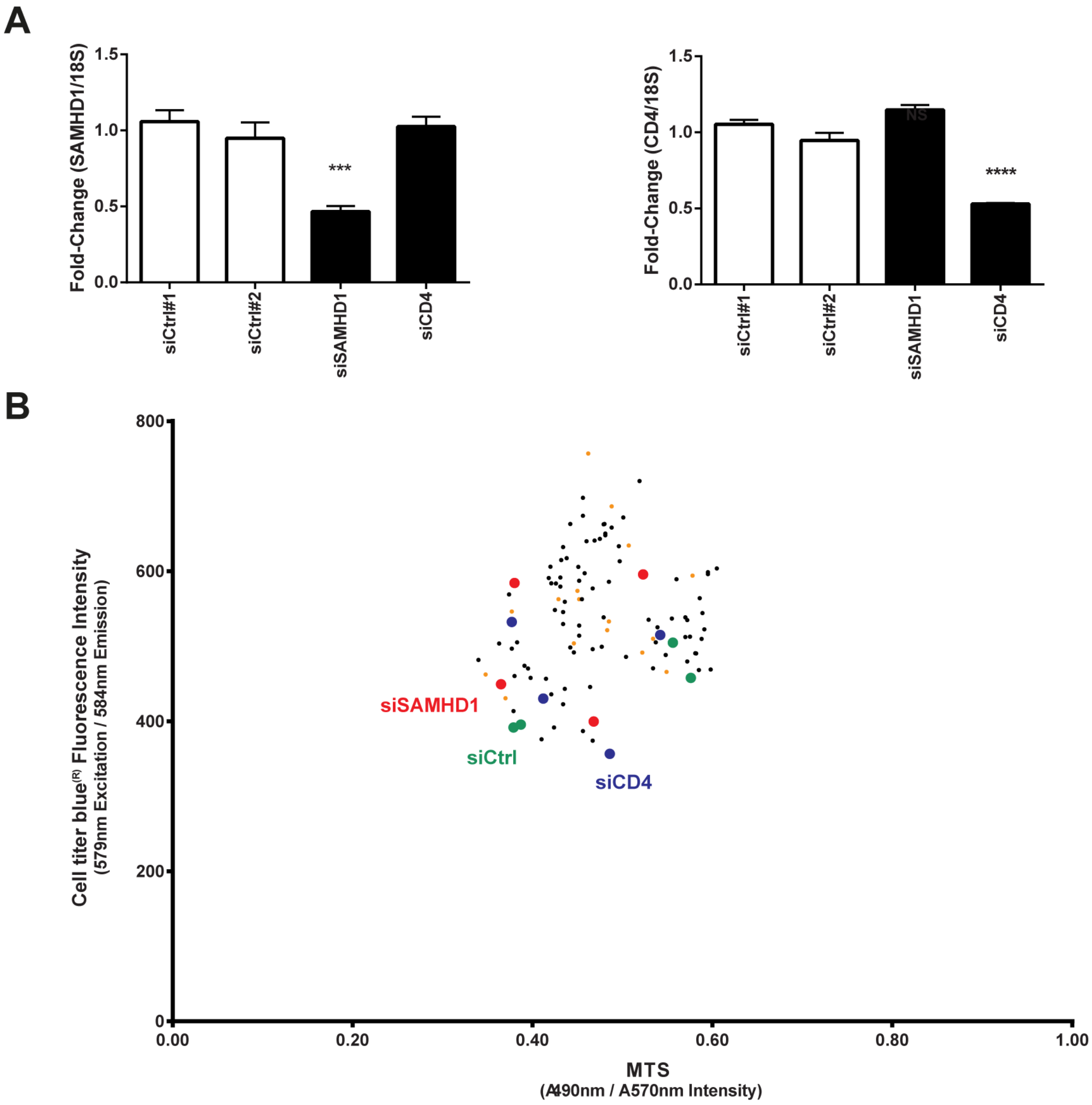
A
Fold-Change (SAMHD1/18S)
1.5
1.0
0.5
0.0
***
siCtrl#1
siCtrl#2
siSAMHD1
siCD4
Fold-Change (CD4/18S)
1.5
1.0
0.5
0.0
NS
****
siCtrl#1
siCtrl#2
siSAMHD1
siCD4
B
Cell titer blue(R) Fluorescence Intensity
(579nm Excitation / 584nm Emission)
800
600
400
200
0
siSAMHD1
siCtrl
siCD4
0.00
0.20
0.40
0.60
0.80
1.00
MTS
(A490nm / A570nm Intensity)

Figure S6

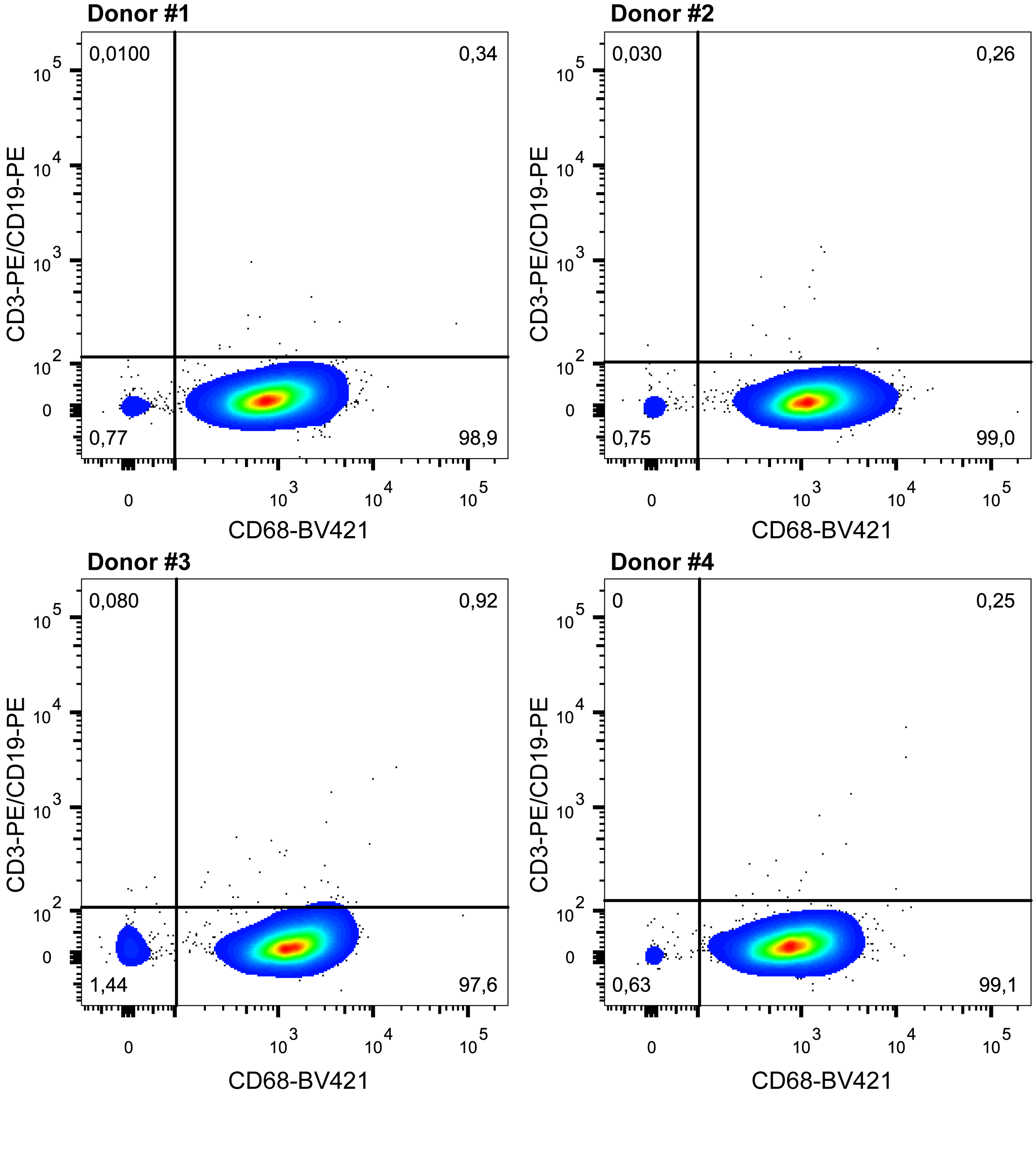

Donor #1
0,0100
0,34
0,77
98,9
CD3-PE/CD19-PE
CD68-BV421
Donor #2
0,030
0,26
0,75
99,0
CD3-PE/CD19-PE
CD68-BV421
Donor #3
0,080
0,92
1,44
97,6
CD3-PE/CD19-PE
CD68-BV421
Donor #4
0
0,25
0,63
99,1
CD3-PE/CD19-PE
CD68-BV421

Figure S7
